# The influence of Polycomb and Trithorax group function on the sexually dimorphic abdomen pigmentation of *Drosophila melanogaster*

**DOI:** 10.1093/g3journal/jkag218

**Published:** 2026-08-07

**Authors:** Devon M Seibert, Ashley V Williams, Madeline A Ranly, Melissa E Williams, Claire C Konys, Michael L Weinstein, Jenna R Rock, Rachel M Stanojev, Corinne R Stone, Emily B Daniel, Eliana G Williams, Mark Rebeiz, Thomas M Williams

**Affiliations:** Department of Biology, University of Dayton, 300 College Park, Dayton, OH 45469, United States; Department of Biology, University of Dayton, 300 College Park, Dayton, OH 45469, United States; Department of Biology, University of Dayton, 300 College Park, Dayton, OH 45469, United States; Department of Biology, University of Dayton, 300 College Park, Dayton, OH 45469, United States; Department of Biology, University of Dayton, 300 College Park, Dayton, OH 45469, United States; Department of Biology, University of Dayton, 300 College Park, Dayton, OH 45469, United States; Department of Biology, University of Dayton, 300 College Park, Dayton, OH 45469, United States; Department of Biology, University of Dayton, 300 College Park, Dayton, OH 45469, United States; Department of Biology, University of Dayton, 300 College Park, Dayton, OH 45469, United States; Department of Biology, University of Dayton, 300 College Park, Dayton, OH 45469, United States; Department of Biology, University of Dayton, 300 College Park, Dayton, OH 45469, United States; Department of Biological Sciences, University of Pittsburgh, Pittsburgh, PA 15260, United States; Department of Biology, University of Dayton, 300 College Park, Dayton, OH 45469, United States

**Keywords:** Polycomb, Trithorax, chromatin, Drosophila, gene regulatory network, *cis*-regulatory element, Hox, bric-à-brac

## Abstract

Animal traits develop through intricate patterns of gene expression that are regulated at multiple levels. This regulation includes interactions between transcription factors and *cis*-regulatory element DNA sequences and the dynamic accessibility of *cis*-regulatory elements due to chromatin modifications and remodeling. Polycomb group and Trithorax group genes are evolutionarily conserved regulators of chromatin state. Although the Polycomb group and Trithorax group genes have well understood roles in developmental gene regulation, the extent to which these complexes contribute to trait evolution remains unclear. Here, we performed a genetic screen to understand how Polycomb group and Trithorax group genes shape the rapidly evolving gene regulatory network responsible for the dimorphic abdomen tergite pigmentation of *Drosophila melanogaster* fruit flies. A near-comprehensive screen of Trithorax group and Polycomb group genes was conducted using RNAi, and numerous genes were identified whose reduced expression caused alterations to tergite pigmentation. For the 8 most impactful genes, their roles in regulating this gene regulatory network were explored. We assessed their effects on several key transcription factors and downstream *cis*-regulatory elements that are responsible for the expression of the gene regulatory network's pigmentation enzyme genes. We show that multiple members of the Polycomb group and Trithorax group complexes are required for distinct tiers in the pigmentation gene regulatory network, suggesting potential points where responsive elements for different factors may have evolved. The results set the stage for future studies to identify the direct gene regulatory network targets of Polycomb group and Trithorax group complexes and how they have participated in the evolution of this gene regulatory network.

## Introduction

The development of animal traits is governed by the action of gene regulatory networks (GRNs) which control differential gene activity (Davidson 2006). Such GRNs rely upon the interactions of transcription factor proteins with their target binding site DNA sequences in *cis*-regulatory elements (CREs) that combinatorially determine the timing, levels, and spatial patterns of gene expression (Bonn and Furlong 2008). As a result, the elucidation of GRNs is an ongoing priority in order to understand trait development and its evolution (Carroll 2008). Studies of trait evolution have rarely focused on the roles of specific chromatin modifiers, in favor of studying the transcription factors which spatially pattern gene activity. Elucidating the roles of chromatin modifiers in rapidly evolving GRNs is thus an important prerequisite to tracing how they participate in GRN evolution.

The evolutionarily conserved Polycomb group (PcG) genes encode proteins that modify histones and remodel chromatin to facilitate the repression of gene expression (Schuettengruber et al. 2007). Conversely, the evolutionarily conserved Trithorax group (TrxG) genes encode proteins that reciprocally modify chromatin to facilitate activation (Kingston and Tamkun 2014). Many of these genes have been identified and the functions of their encoded proteins have been elucidated in the model organism *Drosophila melanogaster* (Kassis et al. 2017). One ideal trait to study is the sexually dimorphic pattern of dorsal abdomen tergite pigmentation of *D. melanogaster* (Fig. 1a), for which the GRN is increasingly well understood at the levels of transcription factors and CREs (Williams et al. 2008; Rebeiz et al. 2009; Rogers et al. 2014; Camino et al. 2015; Grover et al. 2018; Roeske et al. 2018; Hughes et al. 2023; Brubaker et al. 2025). Female *D. melanogaster* are pigmented on the posterior region of the tergites that cover the A2 to A6 abdominal segments. While a similar pattern of tergite pigmentation occurs on the A2 to A4 tergites of males, the A5 and A6 tergites are fully melanic (Fig. 1a). Moreover, this trait and its GRN have evolved dramatically among the *Sophophora* subgenus, including the gain, modification, and repeated loss of this trait (Jeong et al. 2006; Hughes et al. 2020).

**Fig. 1.**
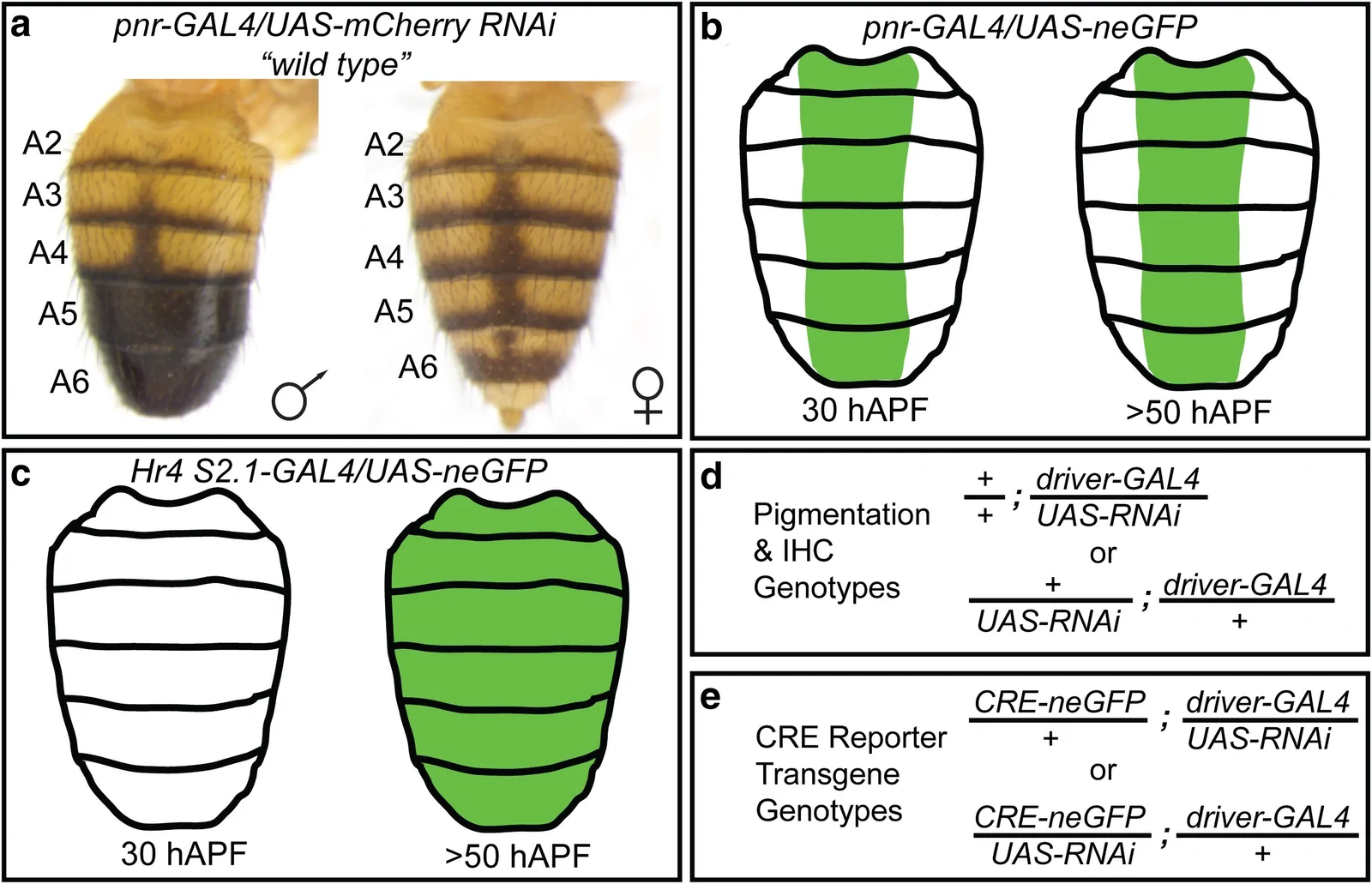
The wild-type *D. melanogaster* abdomen and the genetic details of perturbation experiments used in this study. a) Four-day old male and female abdomens of specimens in which the negative control *mCherry* gene was targeted by RNAi. These phenotypes are considered to be wild type. b) Schematic of the temporal (hAPF) and spatial (dorsal abdomen epidermis) patterns of expression for neGFP when activated by the *pnr-GAL4* allele. c) Schematic of the temporal and spatial patterns of expression for neGFP when activated by the *Hr4 S2.1-GAL4* transgene. d) Genotypes of individuals whose adult dorsal abdomen phenotypes or pupal-stage protein expression patterns (revealed by immunohistochemistry or IHC) were obtained. e) Genotypes of pupae or adults (for *ebony* transgene) in which CRE-neGFP reporter transgene activities were assessed that had a PcG or TrxG gene targeted by RNAi.

The development of black, brown, and yellow pigments that color tergites require spatial- and sex-specific expression patterns for a pathway of “realizator” genes, whose expression-regulating CREs have been identified. Among these CREs are the *MEE1* of *Ddc* (Grover et al. 2018), the *t_MSE2* of *tan*, and the *yBE0.6* of *yellow* (Camino et al. 2015). While more than a dozen transcription factors have been identified that are required for the development of this dimorphic trait (Rogers et al. 2014; Petrosky et al. 2024), 4 stand out for their importance. First, the *Hox* genes *abdominal-A* (*abd-A*) and *Abdominal-B* (*Abd-B*) provide positional segment-specific information along the anterior-posterior axis. Abd-A protein is necessary for the formation of black pigments (Rogers et al. 2014), and it has been shown to interact with binding sites in the *t_MSE2* (Camino et al. 2015). Abd-B protein is similarly necessary for the unique melanic coloration of the A5 and A6 tergites (Kopp et al. 2000) and has been shown to shape the expression of multiple target genes through its influence on the *MEE1*, *t_MSE2*, and *yBE0.6* (Jeong et al. 2006; Camino et al. 2015; Grover et al. 2018). The 2 other key transcription factors in this GRN are the tandem duplicate *bab1* and *bab2* genes. Their encoded transcription factors (Bab1 and Bab2) are expressed in the same dimorphic pattern (Kopp et al. 2000; Salomone et al. 2013; Hughes et al. 2020), where males lack expression in the fully melanic A5 and A6 segments, but there is expression uniformly throughout the female abdomen. This dimorphic pattern of expression is necessary for these proteins to suppress melanic pigmentation in the female abdomen (Roeske et al. 2018). How the PcG and TrxG genes contribute to the dimorphic pigmentation phenotype and the expression patterns of the GRN's key realizator and transcription factor genes has received little attention to date.

In this study, we used RNA-interference (RNAi) to deplete the expression of PcG and TrxG genes one-by-one in order to observe their accompanying phenotypes. The expressions of Abd-A, Abd-B, and Bab1 were then analyzed in genetic backgrounds in which select PcG and TrxG genes were depleted. Additionally, the activities of select pigmentation GRN CREs were evaluated in *nuclear-localized enhanced Green Fluorescent Protein* (*neGFP*) reporter transgene assays in the same RNAi genetic backgrounds, revealing distinct dependencies throughout the pigmentation GRN.

## Methods

### Fly stocks and genetic crosses

The *D. melanogaster* fly stocks used in this study were maintained at 25 °C on a sugar food medium whose recipe was previously described (Salomone et al. 2013). The RNAi stocks used in this study (Tables 1 and 2) were created by the Transgenic RNAi Project (TRiP) at Harvard Medical School (Hu et al. 2021). These RNAi transgenes were integrated into the *att*P2 site on chromosome 3 or the *att*P40 site on chromosome 2. RNAi stocks were purchased from the Bloomington Drosophila Stock Center (BDSC). The *pnr-GAL4* driver (Calleja et al. 2000) stocks used in this study were *pnr-GAL4/TM6B* (BDSC #58788) and *pnr-GAL4/TM3* (BDSC #3039). The *Hr4 S2.1-GAL4* driver was created for this study. The *Hr4 S2.1* CRE was previously shown to drive broad reporter expression during pupal development (Weinstein et al. 2023). This sequence was cloned into the *Asc*I and *Sbf*I sites of a custom-made *GAL4* vector that has the *DsRed* protein-coding sequence under the transcriptional control of the 3XP3 enhancer to drive expression in eyes and ocelli for transgenic line identification (Hughes et al. 2023). This pREP-Hr4_S2.1-GAL4 (Supplementary Document 1) vector was created by GenScript Biotech. This *Hr4 S2.1*-*GAL4* transgene was inserted into the *D. melanogaster att*P2 site by Best Gene Incorporated.

**Table 1.** Summary of the observed phenotypes elicited by RNAi that targeted PcG complex genes.

Gene	BDSC#	Complex	Function	Tergite phenotypes
*Pc*	31110	PRC1	H3K27me3 binding	♀ A6 loss
*Pc*	33964	PRC1	H3K27me3 binding	♂ A4 gain, ♀ A5 gain, ♂ and ♀ cleft
*Pc*	36670	PRC1	H3K27me3 binding	Not noteworthy
*ph-d*	31190	PRC1	PRC1 subunit	♀ A6 loss
*ph-d*	63018	PRC1	PRC1 subunit	Not noteworthy
*ph-p*	31608	PRC1	PRC1 subunit	Not noteworthy
*ph-p*	33669	PRC1	PRC1 subunit	Not noteworthy
*Psc*	31611	PRC1/dRAF	Chromatin binding	Not noteworthy
*Psc*	35297	PRC1/dRAF	Chromatin binding	Not noteworthy
*Psc*	38261	PRC1/dRAF	Chromatin binding	Not noteworthy
*Sce*	31612	PRC1/dRAF	H2A K118 monoubiquitination	Not noteworthy
*Sce*	67924	PRC1/dRAF	H2A K118 monoubiquitination	♂ and ♀ cleft, ♀ A6 loss
*Caf1/NURF 55*	31714	PRC2	Histone binding	Not noteworthy
*Caf1/NURF 55*	34069	PRC2	Histone binding	Not noteworthy
*E(z)*	33659	PRC2	Subunit for H3K27 methylation	♂ and ♀ cleft
*E(z)*	36068	PRC2	Subunit for H3K27 methylation	♂ and ♀ cleft
*Esc*	31618	PRC2	Chromatin opening repression	Not noteworthy
*Su(z)12*	31191	PRC2	H3K27 trimethylation	Not noteworthy
*Su(z)12*	33402	PRC2	H3K27 trimethylation	Not noteworthy
*Jing*	27024	PRC2-assocciated	Transcription factor	♂ A5 loss, ♀ A6 loss, ♀ cleft
*Jing*	35750	PRC2-assocciated	Transcription factor	♂ A5 loss, ♀ A6 loss, ♂ and ♀ cleft
*Pcl*	31189	PRC2-assocciated	Chromatin binding	Not noteworthy
*Pcl*	33945	PRC2-assocciated	Chromatin binding	Not noteworthy
*Pho*	35206	PhoRC	Transcription factor	Not noteworthy
*Pho*	42926	PhoRC	Transcription factor	♂ A4 gain, ♀ A6 loss
*Sfmbt*	32473	PhoRC	Chromatin binding	♂ A4 gain, ♀ A6 loss, ♀ cleft
*Phol*	64945	PhoRC	Transcription factor	♀ A6 loss
*Asx*	31192	PR-DUB	H2A de-ubiquitination	Not noteworthy
*Asx*	51677	PR-DUB	H2A de-ubiquitination	♂ A4 loss, ♀ A6 loss
*kdm2*	33699	dRAF	H3K36 de-methylation	♀ A6 loss
*kdm2*	31360	dRAF	H3K36 de-methylation	♀ A6 loss
*Scm*	31614	Complex connector	Chromatin binding	Not noteworthy
*Scm*	35389	Complex connector	Chromatin binding	♀ A6 loss and cleft

**Table 2.** Summary of the observed phenotypes elicited by RNAi that targeted TrxG complex genes.

Gene	BDSC#	Complex	Function	Tergite phenotypes
*Sbf1*	32419	TAC1	Chromatin binding	Not noteworthy
*nej (CBP)*	27724	TAC1	Histone acetyltransferase	Not noteworthy
*nej (CBP)*	36682	TAC1	Histone acetyltransferase	♀ A6 gain
*Trx*	31092	TAC1, dCOMPASS-like	H3K4 methyltransferase	♂ and ♀ A3-A6 loss
*Trx*	33703	TAC1, dCOMPASS-like	H3K4 methyltransferase	♂ and ♀ A3-A6 loss, ♂ and ♀ cleft
*Mnn1*	31229	dCOMPASS-like	Chromatin binding	♀ A6 loss
*Mnn1*	35150	dCOMPASS-like	Chromatin binding	♀ A6 loss
*Ptip*	31714	dCOMPASS-like	H3K4 methyltransferase	♀ A6 loss
*Ptip*	35269	dCOMPASS-like	H3K4 methyltransferase	♀ A6 loss
*Trr*	29563	dCOMPASS-like	H3K4 tri-methyltransferase	♀ A6 loss, ♀ A3-A4 gain
*Trr*	36916	dCOMPASS-like	H3K4 tri-methyltransferase	♀ A6 loss, ♀ A3-A4 gain
*Ncoa6*	34964	dCOMPASS-like	H3K4 methyltransferase	Not noteworthy
*Utx*	34076	dCOMPASS-like	H3K27 de-methylase	Not noteworthy
*Dpy30L1*	41946	dCOMPASS	H3K4 methyltransferase	Not noteworthy
*Hcf*	32453	dCOMPASS	Chromatin binding	Not noteworthy
*Hcf*	36799	dCOMPASS	Chromatin binding	Not noteworthy
*Set1*	33704	dCOMPASS	H3K4 methyltransferase	Not noteworthy
*Set1*	38368	dCOMPASS	H3K4 methyltransferase	♀ A6 loss, ♀ A6 cleft
*ash2*	35388	dCOMPASS	H3K4 methyltransferase	♀ A6 loss
*ash2*	64942	dCOMPASS	H3K4 methyltransferase	♀ A6 loss, ♀ A6 cleft
*ash1*	33705	ASH1	H3K4 tri-methyltransferase	♂ and ♀ A3-A6 loss
*ash1*	36130	ASH1	H3K4 tri-methyltransferase	♂ and ♀ A3-A6 loss
*Bap55*	31708	BAP, SWI-SNF	Chromatin remodeling	♀ A6 loss
*e(y)3*	32346	BAP, SWI-SNF	Chromatin binding	Not noteworthy
*Mor*	35630	BAP, SWI-SNF	Histone binding	Not noteworthy
*Mor*	35662	BAP, SWI-SNF	Histone binding	♀ A6 loss
*Osa*	31266	BAP, SWI-SNF	DNA binding	♂ and ♀ A3-A6 loss, ♂ cleft
*E(bx)*	31193	NURF	Histone binding	♀ A6 loss
*E(bx)*	35353	NURF	Histone binding	♀ A6 loss
*Iswi*	32845	NURF	ATP utilization	Not noteworthy
*Iswi*	51931	NURF	ATP utilization	♀ A6 loss
*Nurf*	35444	NURF	Phosphatase activity	♀ A6 loss
*Chd1*	34665	CHD	ATPase	♀ A6 loss
*Chd1*	35240	CHD	ATPase	♀ A6 loss
*Kis*	34908	Kis	Chromodomain trans. factor	♂ A3-A6 loss and ♀ A6 loss
*Kis*	36597	Kis	Chromodomain trans. factor	♂ A3-A6 loss and ♀ A6 loss
*Kis*	44542	Kis	Chromodomain trans. factor	♂ A3-A6 loss and ♀ A6 loss
*Kto*	34548	Mediator	Kinase	♂ and ♀ A6 loss
*Kto*	80447	Mediator	Kinase	♂ and ♀ cleft, ♀ A5-A6 gain
*Mi-2*	33419	CHD	Nucleosome remodeler	♂ A3-A4 gain, ♀ A5-A6 gain

To test the spatiotemporal activity of the GAL4 drivers, virgin female *UAS-neGFP* (BDSC #4776) flies were crossed to males from the *GAL4* driver lines, creating F1s with the needed dihybrid genotypes (Fig. 1b and c). To perform *UAS-RNAi* experiments, virgin female *pnr-GAL4/TM3* flies were crossed to *UAS-RNAi* transgene line males to obtain progeny with the needed dihybrid genotypes (Fig. 1d). To assess transcription factor expression patterns in an *RNAi* genetic background, *pnr-GAL4/TM6B* virgin females were crossed to males from the *UAS-RNAi* transgene lines to obtain progeny with the needed dihybrid genotype (Fig. 1d).

To test *RNAi* transgenes for effects on *CRE-neGFP* reporter transgenes, fruit flies were created with the genotypes *CRE-neGFP* (in either the *att*P40 or 51D *att*P site on chromosome 2); *pnr-GAL4/TM6B*. The *CRE-neGFP* transgenes used were *bab dimorphic element-neGFP* (Rogers et al. 2013), *Ddc MEE1-neGFP* (Grover et al. 2018), *tan t_MSE2-neGFP*, and *yellow yBE0.6-neGFP* (Camino et al. 2015). The *t_MSE2* and *MEE1* reporter transgenes were inserted into the second chromosome 51D *att*P site (Bischof et al. 2007), while the *dimorphic element* and *yBE0.6* transgenes were inserted into the second chromosome *att*P40 site (Markstein et al. 2008). Virgin females from these *GAL4* driver and *CRE-neGFP* reporter stocks were crossed to males from the *UAS-RNAi* stocks in order to obtain progeny with the needed genotypes (Fig. 1e).

### Pupal abdomen immunohistochemistry

*D. melanogaster* specimens were dissected to collect the dorsal abdomens of pupae at various times after the initiation of pupal development, so-called hours after puparium formation or hAPF. These times were based upon *D. melanogaster* development at 25 °C in which the different developmental stages can be distinguished by the presence and absence of certain morphological characters (Ashburner et al. 2011; Grover et al. 2018; Hughes et al. 2020).

The immunohistochemistry procedure was performed on male and female specimens that were combined in the same tubes, such that the same conditions were experienced. Specimens were fixed for 35 min in a PBST solution (phosphate-buffered saline with 0.3%Triton X-100) that included 4% paraformaldehyde (Electron Microscopy Services). Specimens were rinsed twice in PBST, then washed twice in PBST for 5 min, and then transferred into PBST supplemented with 1% bovine serum albumin (the blocking solution) and incubated for 1 h at room temperature. The specimens were then transferred into PBST with a primary antibody (anti-Bab1) or antibodies (anti-Abd-A and anti-Abd-B), and incubated overnight at 4 °C. The primary antibodies used were a 1:200 dilution of the affinity purified rabbit-anti-Bab1 antibody (Williams et al. 2008), 1:500 dilution of guinea pig anti-Abd-A antibody (Li-Kroeger et al. 2008; Rogers et al. 2014), and a 1:200 dilution of the mouse monoclonal antibody (Developmental Studies Hybridoma Bank, 1A2E9) against *D. melanogaster* Abd-B (Weinstein et al. 2023).

Following primary antibody incubation, the specimens were rinsed twice in PBST, then washed twice in PBST for 5 min, and then transferred into blocking solution for a 1-h incubation. The specimens were next incubated with a 1:500 dilution of 1 (Bab1 specimens) or 2 (Abd-A and Abd-B specimens) Alexa Fluor secondary antibodies. These were goat anti-rabbit Alexa Fluor 647 (Invitrogen, #A21244) in PBST for Bab1, goat anti-mouse 647 (Invitrogen, #A21236) for Abd-B, and goat anti-Guinea Pig 488 (Invitrogen, #A11073) for Abd-A. The specimens were incubated with secondary antibody for 4 h at room temperature. After this incubation, specimens were washed for 5 min in PBST 4 times and then allowed to equilibrate for 15 min in the glycerol mount (50% glycerol, 50% PBST) solution. After equilibration, the specimens were moved to the glycerol mount (now 80% glycerol) and then placed between a glass slide and coverslip. Cover slips and slides were attached by a piece of double-sided sticky tape with a central hole cut out by a razor blade. These specimens were positioned in the hole with the cuticle side up (adjacent to the cover slip) and the epidermis side down (toward the slide surface).

### Imaging specimens for gene expression patterns and pigmentation phenotypes

Images of *D. melanogaster* adult dorsal abdomen pigmentation patterns were taken by an Olympus SZX16 Zoom Stereoscope with a DP72 digital camera. The camera was controlled by the Olympus CellSens Standard 2.2 software package. Specimens were imaged at a 50× magnification. RNAi cross progeny were aged to 4 d old. At which time, their legs, wings, and head were removed and then the bodies were mounted on double-sided tape that was adhered to a conventional glass microscope slide. Images were taken of these situated specimens. Images of the dorsal thorax were taken for some but not all evaluated *RNAi* transgenes. Thus, the absence of thorax data does not indicate that the RNAi transgene lacked an effect on the thorax.

An Olympus Fluoview FV 1000 confocal microscope and software was used to take projection images for the *neGFP* reporter transgene expressions in *D. melanogaster*. Specimens were imaged at pupal developmental stages when the CRE was for either *bab*, *Ddc*, *tan*, or *yellow*. For pupal-stage samples, pupae were removed from the puparium and then placed in a drop of halocarbon 700 oil on standard glass microscope slides. Generic settings for imaging neGFP expression patterns were laser 30% power (488 laser), gain set to 1, offset set to 1, Kalman line averaging set to 2, aperture set to 200 microns, and z-series step size set to 10 microns. The HV values used were 650 for the *Ddc MEE1* specimens, 675 for *bab dimorphic element* specimens, 690 for *yBE0.6* specimens, and 725 for *t_MSE2* specimens.

Images of immunohistochemistry specimens were also taken with a Fluoview FV 1000 confocal microscope and software. Abd-A expressions were taken using the 633 nm laser at 30% power. Abd-B and Bab1 expression were taken using the 488 nm laser at 30% power. The HV settings used were 650 for Abd-A, 800 for Abd-B, and 725 for Bab1. All immunohistochemistry images were taken with the gain set to 1, offset set to 1, Kalman line averaging set to 2, aperture at 200 microns, and z-series step size set to 2 microns when using the 20× objective and 5 microns when using the 10× objective.

### Figure development

All neGFP and immunohistochemistry expression patterns shared were representative images from replicate specimens of the same genotype. All abdomen pigmentation phenotypes and transgene reporter expression were replicated in 3 or more individuals. Protein expression patterns as revealed by immunohistochemistry were replicated in 2 or more individuals. Images with the same genotypes in a main figure and a supplementary figure are replicate specimens. TIFF images of neGFP expression patterns were processed through the same sequences of modifications using Adobe Photoshop CS3. Likewise, the same set of modifications was done for TIFF images of adult pigmentation patterns and for immunohistochemistry images. Figures were assembled using Adobe Illustrator CS3.

## Results

### RNAi screen for PcG and TrxG abdominal pigmentation phenotypes

In order to identify the PcG and TrxG components required for wild-type *D. melanogaster* pigmentation, we performed an initial screen using the *pnr-GAL4* chromosome, which drives dorsal midline expression from embryonic stages (Calleja et al. 2000) through early and late pupal development (Supplementary Fig. 1). In our experience, the available RNAi transgenes that target important pleiotropic developmental loci in conventional *att*P insertion sites are often those that elicit weak expression knockdowns that allow transgenic stocks to be viable (Rogers et al. 2014; Roeske et al. 2018; Brubaker et al. 2025). To reduce the risk of false negative outcomes, we screened multiple unique RNAi lines when available that target the same gene. Furthermore, we have found short-hairpin RNAi transgenes to be specific to their targeted locus (Rogers et al. 2014; Grover et al. 2018; Weinstein et al. 2023; Brubaker et al. 2025). Thus, we consider interesting phenotypes hailing from a single RNAi transgene worth reporting and likely to be specific to the targeted gene, though further validation is warranted beyond this initial report. The phenotypes of RNAi knockdown were compared to *mCherry* RNAi, which served as a negative control due to the absence of this gene in the *D. melanogaster* genome (Fig. 2a).

**Fig. 2.**
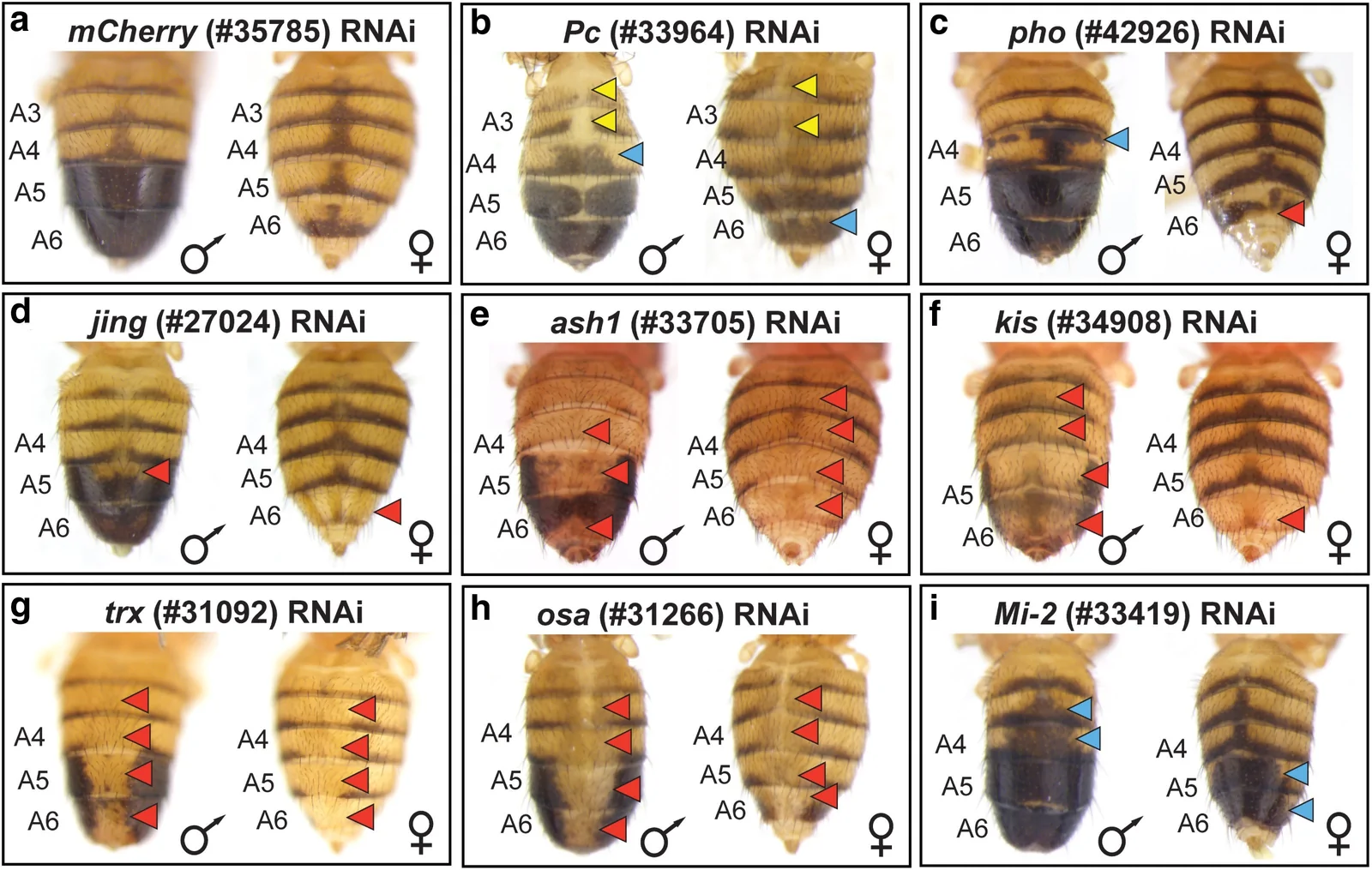
Conspicuous abdomen tergite pigmentation phenotypes from RNAi for certain PcG and TrxG genes. All *UAS-RNAi* transgenes were ectopically expressed along the dorsal abdomen midline region under the regulation of the *pnr-GAL4* driver. The Bloomington Drosophila Stock Center lines used for the RNAi transgenes are listed in parentheses. a) The *UAS-mCherry* RNAi transgene is considered a negative control as it targets a gene that does not exist in the *D. melanogaster* genome. b to d) RNAi targeting a gene that encodes a part of a PcG complex. e to i) RNAi targeting a gene that encodes are part of a TrxG complex. Blue arrowheads point to ectopic melanic pigmentation, red arrowheads point to reductions in melanic pigmentation, and yellow arrowheads point to clefts in the abdomen.

Among PcG components, we observed strong phenotypes (Fig. 2b and c) for *Polycomb* (*Pc*) and *pleiohomeotic* (*pho*). *Pc* encodes the major PRC1 subunit that recognizes H3K27me3 marks deposited by PRC2 components via its chromodomain to mediate DNA compaction (Kassis et al. 2017). Notably, expression of the *UAS-Pc RNAi* transgene resulted in ectopic gains in pigmentation on the male A4 and female A5 tergites in one line (Fig. 2b), but subtle or no effects in the other 2 lines tested (Supplementary Fig. 2). We observed a consistent reduction of female A6 pigmentation in *pho* RNAi abdomens, as well as ectopic pigmentation in male A4 (Fig. 2c and Supplementary Fig. 3). *pho* encodes a DNA-binding transcription factor that recruits the PRC1, PRC2, and PR-DUB complexes to Polycomb response elements (PREs).

The PRC2 complex has its activity modified by association with the protein encoded by the *jing* gene (Kang et al. 2015; Kassis et al. 2017). Two independent lines were used to target *jing*, and both resulted in melanic pigmentation losses on the anterior A5 tergite of males and pigmentation loss on the female A6 tergite (Fig. 2d; Supplementary Fig. 4i to l). These findings indicate that the PRC2 complex and its activity modification can shape aspects of abdomen melanic and nonmelanic pigmentation.

The TrxG components *ash1*, *kis*, and *trx* all gave consistent pigmentation phenotypes, including a sharp reduction in A5 and A6 pigmentation in males and reductions of posterior stripes abdomen-wide (Fig. 2e to g; Supplementary Figs. 5 to 7). *trithorax* (*trx*) is the namesake of the TrxG and is responsible for the deposition of histone H3K4me3 marks (Kassis et al. 2017). *kismet* (*kis*) encodes a SNF2-like protein that facilitates chromatin remodeling. *ash1* encodes a protein that functions in H3K36 dimethylation. The strong consistency of these TrxG components motivated us to compare their effects on the pigmentation GRN, presented below.

In addition to histone modifications, TrxG complexes function in chromatin remodeling. One complex is the Brahma Associated Protein (BAP) complex that includes the protein encoded by *osa* gene (Table 2) (Kassis et al. 2017). The Osa protein possesses an A-T rich DNA-binding domain. RNAi targeting *osa* caused a dramatic loss in melanic pigmentation for the A3 to A6 tergites of male and female *D. melanogaster* and a noticeable cleft in the male abdomen (Fig. 2h; Supplementary Fig. 8i and j). One explanation for such a cleft is that it results from a reduced proliferation of adult abdominal histoblast cells and a retention of larval epithelial cells. In such regions, the absence of melanic pigmentation would be secondary to this cellular aberration. Important here, the losses and gains of melanic pigmentation (Fig. 2) predominately occur in regions lacking the cleft phenotype. The NuRD (Nucleosome Remodeling and Deacetylase) complex is a multiprotein complex in cells that is involved in gene expression regulation and chromatin organization (Lai and Wade 2011). NuRD couples ATP-dependent chromatin remodeling and histone deacetylation. The *Mi-2* gene (Table 2) encodes a protein involved in this complex. RNAi targeting *Mi-2* led to ectopic pigmentation on the male A3 and A4 tergites and the female A5 and A6 tergites (Fig. 2i; Supplementary Fig. 6k and l).

A variety of additional PcG and TrxG factors were tested by RNAi. These resulted in little-to-no conspicuous tergite pigmentation phenotypes or in abnormal abdomen and/or thorax development. These results are described in Supplementary Document 2 and Supplementary Figs. 2 to 16.

### Different RNAi drivers implicate some PcG and TrxG genes as acting earlier during tergite development

*D. melanogaster* pupal development unfolds over the course of ∼100 h, and the pigmentation metabolic enzyme genes are known to be expressed at 70 hAPF or later, including *ebony* expression which becomes robust and sexually dimorphic after pupal development is complete (shortly after eclosion). We therefore tested a second driver, *Hr4 S2.1-GAL4*, which is active ∼20 h later during pupal development than the *pnr-GAL4* driver (Supplementary Fig. 1). Our question was whether delayed activation (50 hAPF rather than ≤30 hAPF) would have similar or distinct consequences on the adult abdomen pigmentation phenotypes. We selected the PcG and TrxG genes whose RNAi resulted in the most striking pigmentation phenotypes when driven by the *pnr-GAL4* allele (Fig. 2): *ash1*, *jing*, *kis*, *Mi-2*, *osa*, *Pc*, *pho*, and *trx*. In males, similar pigmentation aberrations were seen when RNAi was activated by *Hr4 S2.1-GAL4* except for *ash1*, *Mi-2*, and *osa* (Fig. 3). In females, the 2 *GAL4* drivers elicited similar phenotypes when RNAi targeted *jing*, *kis*, *Pc*, and *trx* (Fig. 4), though there were some noteworthy differences. While *pnr-GAL4* drove notable ectopic tergite pigmentation in females with *Mi-2* and *pho* RNAi, the *S2.1-GAL4* resulted in tergite pigmentation losses for these genes (Figs. 3 and 4). This suggests distinct temporal dependencies of the GRN on these genes, transitioning from promoting melanic pigmentation early on to an antagonization at later stages.

**Fig. 3.**
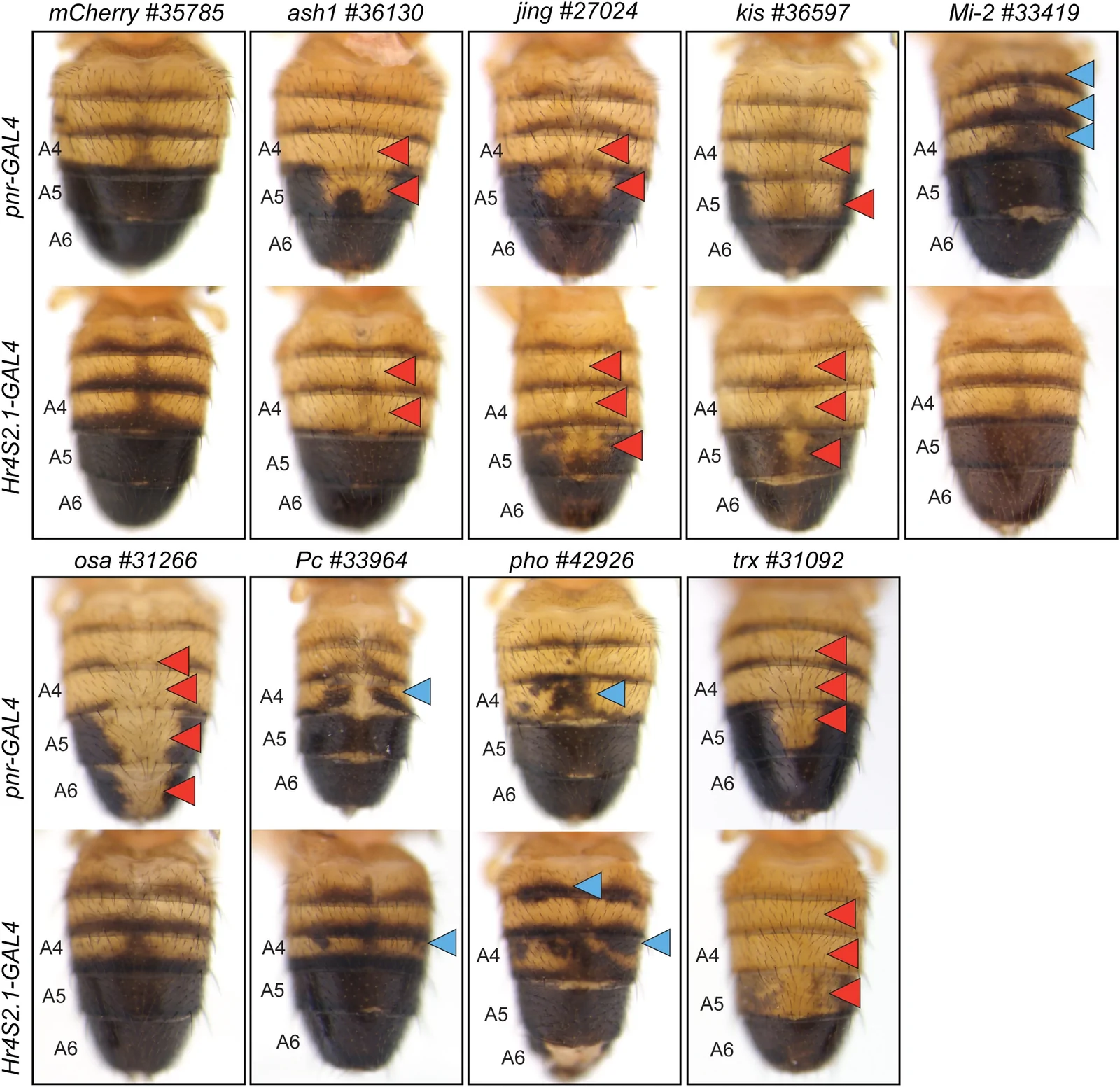
Comparison of RNAi tergite pigmentation phenotypes in *D. melanogaster* males when RNAi was driven by 2 different GAL4 drivers. The GAL4 drivers used were *pnr-GAL4* (top images) and *Hr4 S2.1-GAL4* (bottom images). The listed numbers (#) are the Bloomington Drosophila Stock Center identifiers used for each RNAi transgene. Blue arrowheads point to ectopic melanic pigmentation, while red arrowheads point to reductions in melanic pigmentation. *mCherry* RNAi was used as a negative control. The RNAi transgenes tested were identified originally due to striking phenotypes when ectopically expressed by the *pnr-GAL4* driver. Similar pigmentation phenotypes were observed for *Pc*, *pho*, *jing*, *kis*, and *trx* RNAi. However, far less noticeable phenotypes occurred when RNAi for *ash1*, *osa*, and *Mi-2* were driven by the *Hr4 S2.1-GAL4*.

**Fig. 4.**
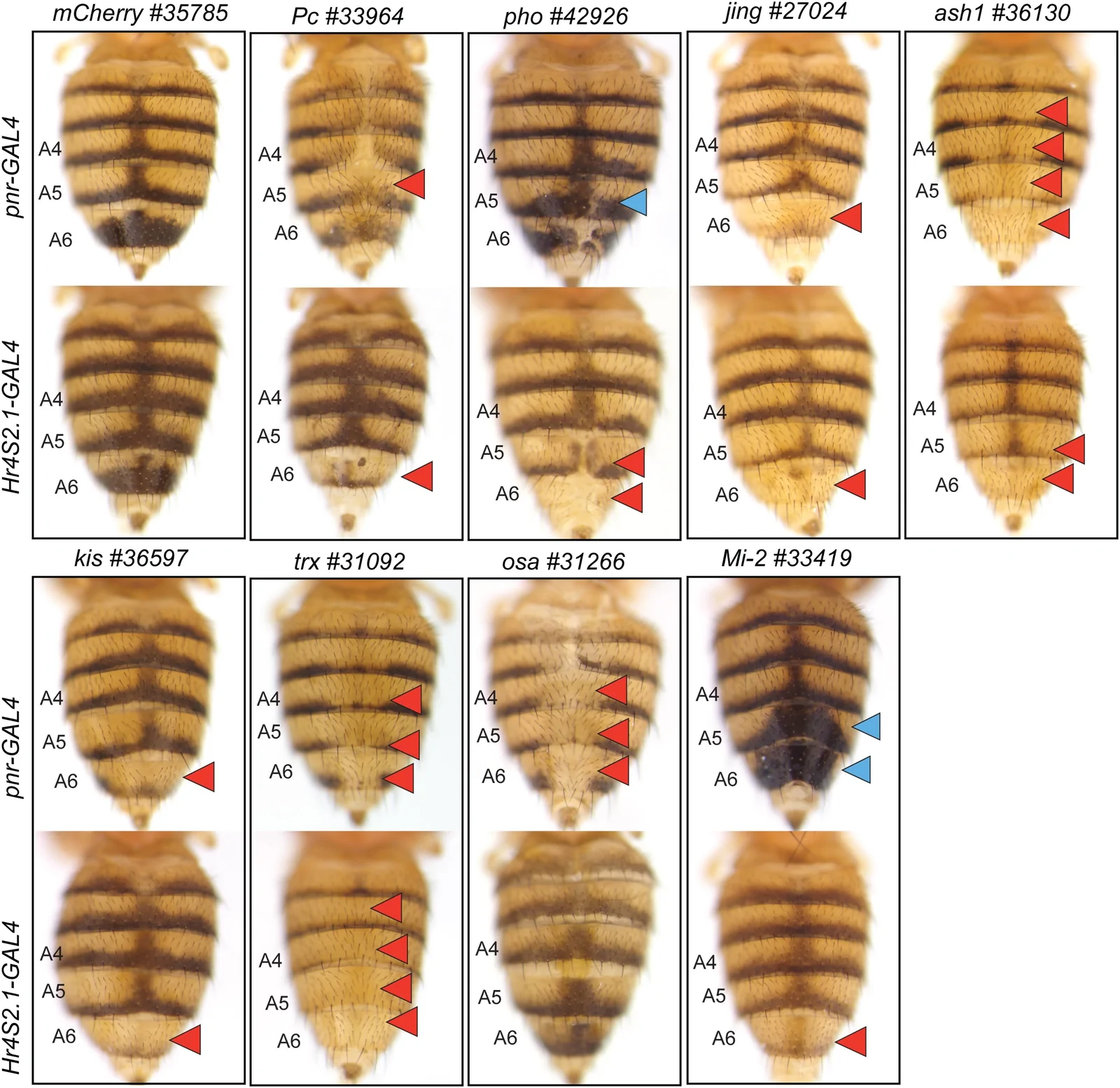
Comparison of RNAi tergite pigmentation phenotypes in *D. melanogaster* females when RNAi was driven by 2 different *GAL4* drivers. The *GAL4* drivers used were *pnr-GAL4* (top images) and *Hr4 S2.1-GAL4* (bottom images). The listed numbers (#) are the Bloomington Drosophila Stock Center identifiers used for each RNAi transgene. Blue arrowheads point to ectopic melanic pigmentation, while red arrowheads point to reductions in melanic pigmentation. *mCherry* RNAi was used as a negative control. The RNAi transgenes tested were identified originally due to striking phenotypes when ectopically expressed by the *pnr-GAL4* driver. Similar pigmentation phenotypes were observed for *Pc*, *pho*, *jing*, *ash1*, *kis*, and *trx* RNAi. However, a noticeable phenotype was absent when RNAi for *osa* and *Mi-2* were driven by the *Hr4 S2.1-GAL4*.

### Interactions between PcG and TrxG genes and key patterning genes of the tergite pigmentation GRN

While PcG and TrxG complexes are essential for the tergite pigmentation characteristic of *D. melanogaster*, it remained unknown how these complexes impact the underlying transcription factors for this GRN. Two key transcription factors are the Hox proteins Abdominal-A (Abd-A) and Abdominal-B (Abd-B). Abd-A is a key determinant of A2 to A4 identity whose expression spans the A2 to A6 segment epidermis throughout pupal development (Kopp and Duncan 2002; Rogers et al. 2014). Abd-B is the key determinant of A5 and A6 identity whose expression occurs in these segments, with expression higher in A6 than A5 (Kopp and Duncan 2002; Weinstein et al. 2023). A previous study showed how Abd-B's segmental extent of expression changed between larval and pupal stages in *D. santomea*, a species closely related to *D. melanogaster* (Liu et al. 2019). To bring temporal clarity on Abd-B expression in *D. melanogaster* during pupal development, we assessed expression at 60 hAPF and at several stages leading up to 95 hAPF. This time window precedes the activation of all the pigmentation realizator genes and continues through the time when the late activated *tan* and *pale* genes become expressed (Camino et al. 2015; Grover et al. 2018; Hughes et al. 2020). We saw that Abd-B expression was continuous during these stages and was comparable between females and males (Supplementary Figs. 17 and 18).

We tested the PcG genes *Pc* and *pho* and the TrxG genes *ash1*, *kis*, *osa*, *Mi-2*, and *trx* for effects on the expression of Abd-A and Abd-B (Supplementary Figs. 19 to 26). Among these conditions, Abd-A expression was only noticeably altered by *ash1* RNAi, where expression was reduced in the A2-A6 tergites (zoom in of A3 expression in Fig. 5b; Supplementary Fig. 20 shows whole abdomen expression). For Abd-B, expression was also reduced in response to *ash1* RNAi, with reduction observable in the A5 (Fig. 6b′) and less so for the A6 (Supplementary Fig. 20) epidermis. Conversely, RNAi for *Mi-2*, *Pc*, and *pho* caused ectopic gains in Abd-B expression in the A4 segment epidermis (Fig. 6d, f, and g; Supplementary Figs. 22, 24, and 25), suggesting *ash1* functions in Hox expression activation and/or maintenance, while the other 3 directly or indirectly facilitate Abd-B repression in the more anterior abdomen segments.

**Fig. 5.**
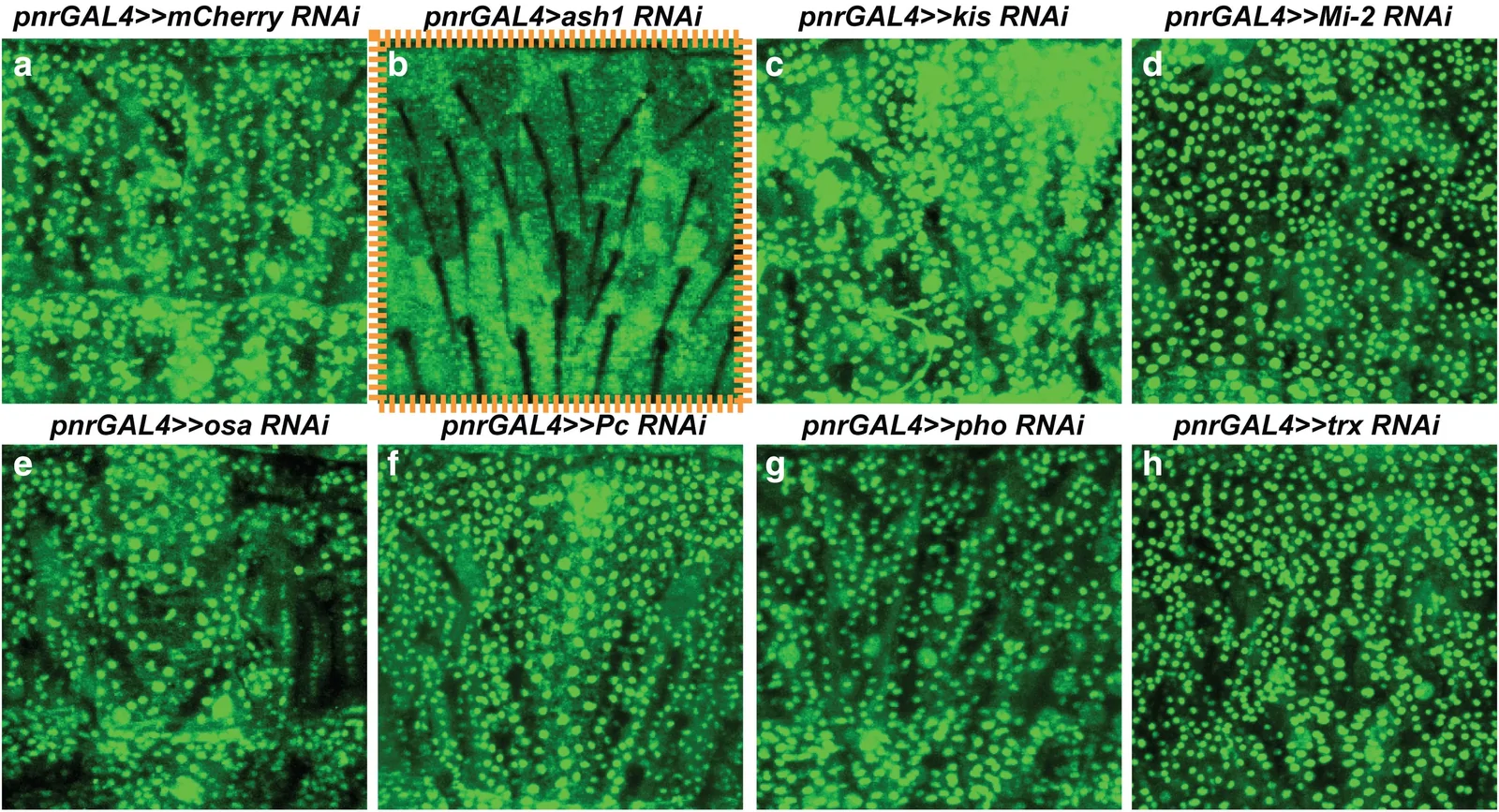
Abd-A expression in the dorsal pupal abdomen epidermis of *D. melanogaster* pupae for which a PcG or TrxG complex gene's expression was reduced by RNAi. a to h) The RNAi transgenes’ expression was driven by the *pnr-GAL4* driver. Specimens were collected at the 85 to 88 hAPF pupal stage for *D. melanogaster*. Epidermal cell expression is seen as the small green circles. a) *mCherry* RNAi was used as the negative control. c to h) While Abd-A expression was generally not appreciably changed (*kis*, *Mi-2*, *osa*, *Pc*, *pho*, and *trx*), b) Abd-A expression was noticeably reduced in cells for which *ash1* expression was targeted. All images are from a midline zone of the A3 segment, and the full image can be found in Supplementary Figs. 19 to 27.

**Fig. 6.**
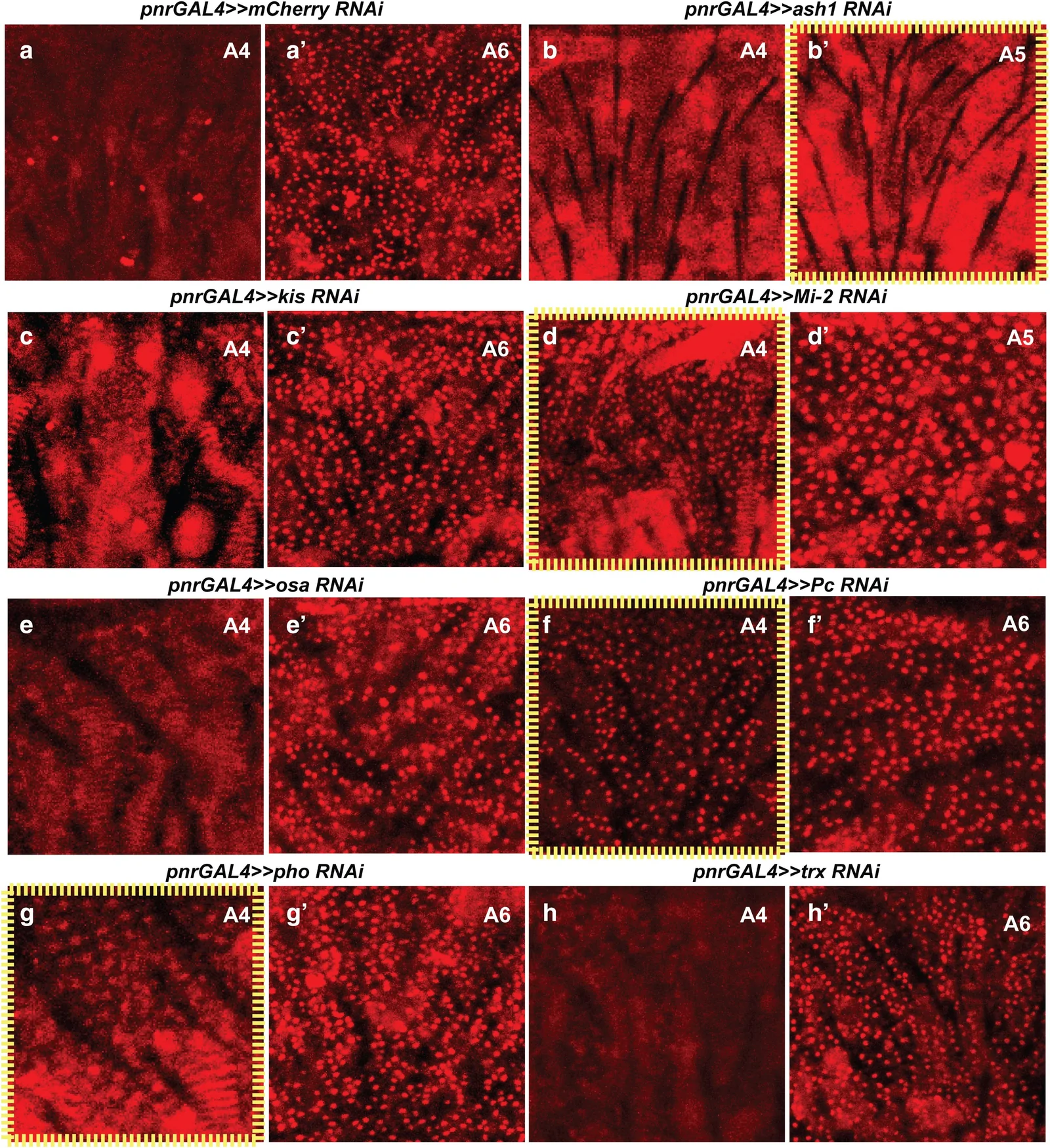
Abd-B expression in the dorsal pupal abdomen epidermis of *D. melanogaster* pupae for which a PcG or TrxG complex gene's expression was reduced by RNAi. a to h and a′ to h′) RNAi transgene expression was driven by the *pnr-GAL4* driver. All specimens were collected at the 85 to 88 hAPF pupal stage for *D. melanogaster*. Epidermal cell nuclei expression is observed as the small red circles. a to h) Images are from a midline zone of the A4 segment. a′ to h′) Images are from the midline zone of the A6 or A5 segment. The full images can be found in (Supplementary Figs. 19 to 27). a and a′) *mCherry* RNAi was used as the negative control, for which Abd-B expression is absent from the A4 segment epidermis and present in the A6 segment epidermis (as well as the A5 segment, not shown here). b′) Abd-B expression was reduced in the A5 segment when *ash1* expression was targeted by RNAi. d, f, and g) Abd-B expression occurred ectopically in the A4 segment epidermis when RNAi targeted *Mi-2*, *Pc*, and *pho* by RNAi.

Two key regulators that shape the sexually dimorphic pattern of *D. melanogaster* tergite pigmentation are the paralogous Bab1 and Bab2 transcription factors. These proteins are expressed in strikingly similar patterns in the pupal abdomen epidermis (Salomone et al. 2013). Female expression occurs throughout the A2 to A6 segment epidermis, while expression in males is noticeably reduced in the A2 to A4 segments and nearly indetectable in the A5 and A6 segments. In this study, we assessed Bab1 expression due to the availability of an antibody (Williams et al. 2008). RNAi for the 7 influential PcG and TrxG genes resulted in no noteworthy alteration to the low level of Bab1 expression that occurs in the male abdomen epidermis (Fig. 7; Supplementary Figs. 19 to 26). In females, expression was generally unaltered, except for a stark reduction in expression that occurred following *Mi-2* RNAi (Fig. 7d; Supplementary Figs. 19 to 26). The influence of Mi-2 on Bab1 expression is consistent with the pigmentation pattern that was observed following *Mi-2* RNAi (Fig. 2i), which approximates the *bab1* and *bab2* RNAi phenotype (Roeske et al. 2018). These results establish the variable nature by which similar phenotypes caused by TrxG and PcG manifest at the *trans*-regulatory tier of the pigmentation GRN. It is important to note that these RNAi-induced alterations on transcription factor expression reveal that particular PcG and TrxG genes function as an upstream regulator. Whether regulation is direct (protein to CRE interaction) or indirect (regulation of an intermediary factor) remains to be resolved.

**Fig. 7.**
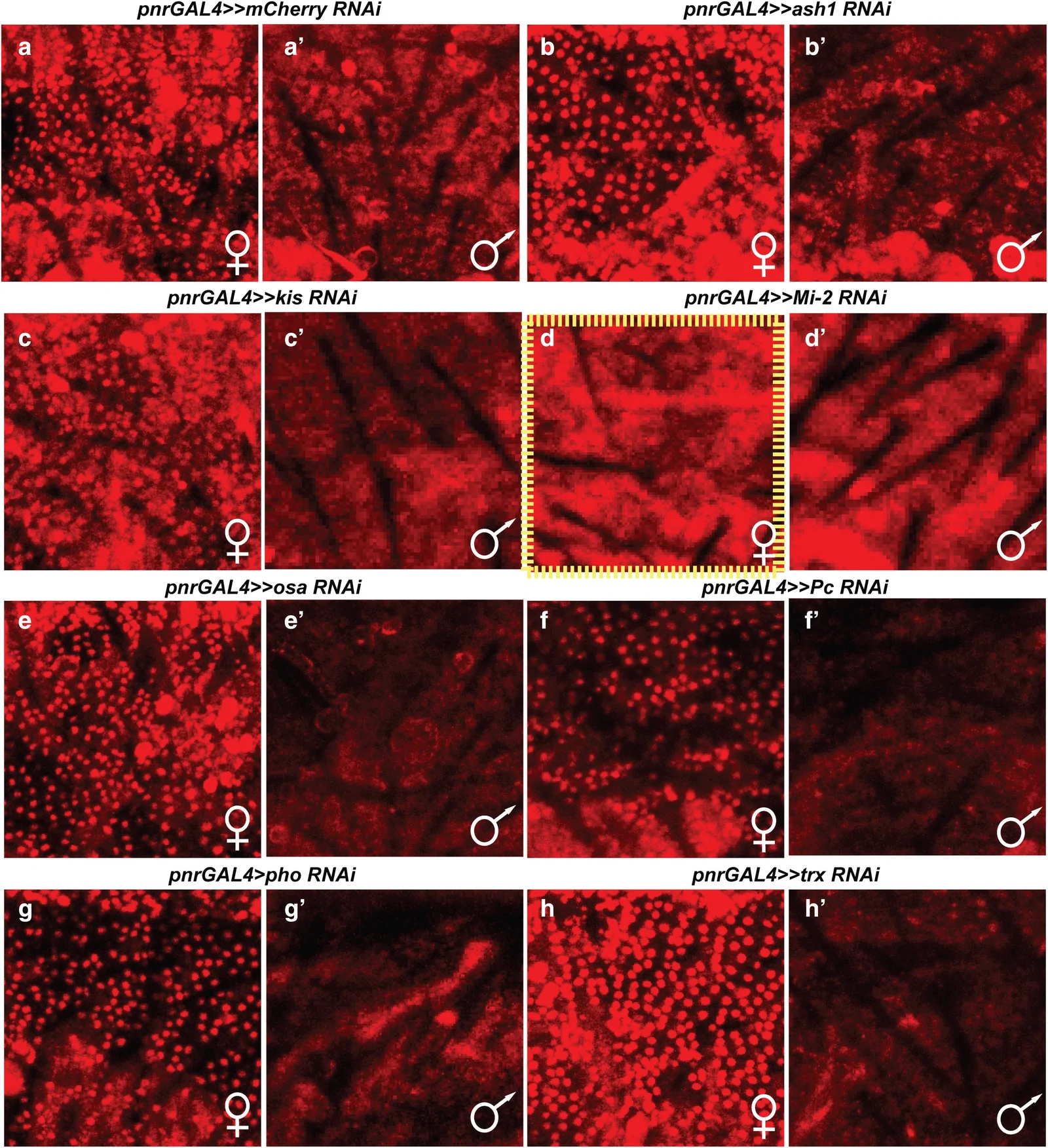
Bab1 expression in the dorsal pupal abdomen epidermis of *D. melanogaster* pupae for which a PcG or TrxG complex gene's expression was reduced by RNAi. a to h and a′ to h′) RNAi transgene expression was driven by the *pnr-GAL4* driver. All specimens were collected at the 85 to 88 hAPF pupal stage for *D. melanogaster*. Epidermal cell nuclei expression is observed as the small red circles. a to h) Images are from a midline zone of the female A6 segment. a′ to h′) Images are from the midline zone of the male A6 segment. The full images can be found in (Supplementary Figs. 19 to 27). *mCherry* RNAi was used as the negative control, for which Bab1 expression occurs in the female A6 segment epidermis but not the male A6 segment. This sexually dimorphic pattern of Bab1 expression was unchanged in samples with b) and b′) *ash1*, c and c′) *kis*, e and e′) *osa*, f and f′) *Pc*, g and g′) *pho*, and h and h′) *trx* RNAi. d and d′) When *Mi-2* was targeted by RNAi, Bab1 expression was reduced in the female A6 segment similar to what occurs in the male A6 segment.

### Interactions between PcG and TrxG genes and CREs controlling *bab* and realizator gene expression

We next sought to determine whether the effects of these perturbations are reflected in the activities of characterized CREs in the pigmentation GRN. The *bab dimorphic element* CRE activates female-specific Bab expression in the dorsal A5 and A6 epidermis (Williams et al. 2008; Rogers et al. 2013). When RNAi against the negative control *mCherry* gene was driven in the dorsal abdomen midline by *pnr-GAL4*, the *dimorphic element* activated neGFP expression robustly in the A6 segment and at a lower level in the A5 segment (Fig. 8a and a′). *Dimorphic element*-driven expression of neGFP was comparable to wild type under RNAi conditions for *ash1*, *jing*, *kis*, and *trx* (Fig. 8). However, RNAi for *Mi-2* and *pho* resulted in increased neGFP in the A5 segment of females, while *osa* RNAi resulted in a reduction in neGFP in the A5 and A6 segments (Fig. 8e to g and e′ to g′). No noticeable differences were observed in the very low level of neGFP expression driven by the dimorphic element in males (Supplementary Fig. 27). These results suggest that Mi-2 and Pho by some means act repressively on *dimorphic element* activity, while Osa acts to promote this CRE's activity. Perhaps reduced *dimorphic element* activity is compensated for in vivo by the activity of the *anterior element* CRE that controls *bab* expression in the anterior abdomen (Williams et al. 2008).

**Fig. 8.**
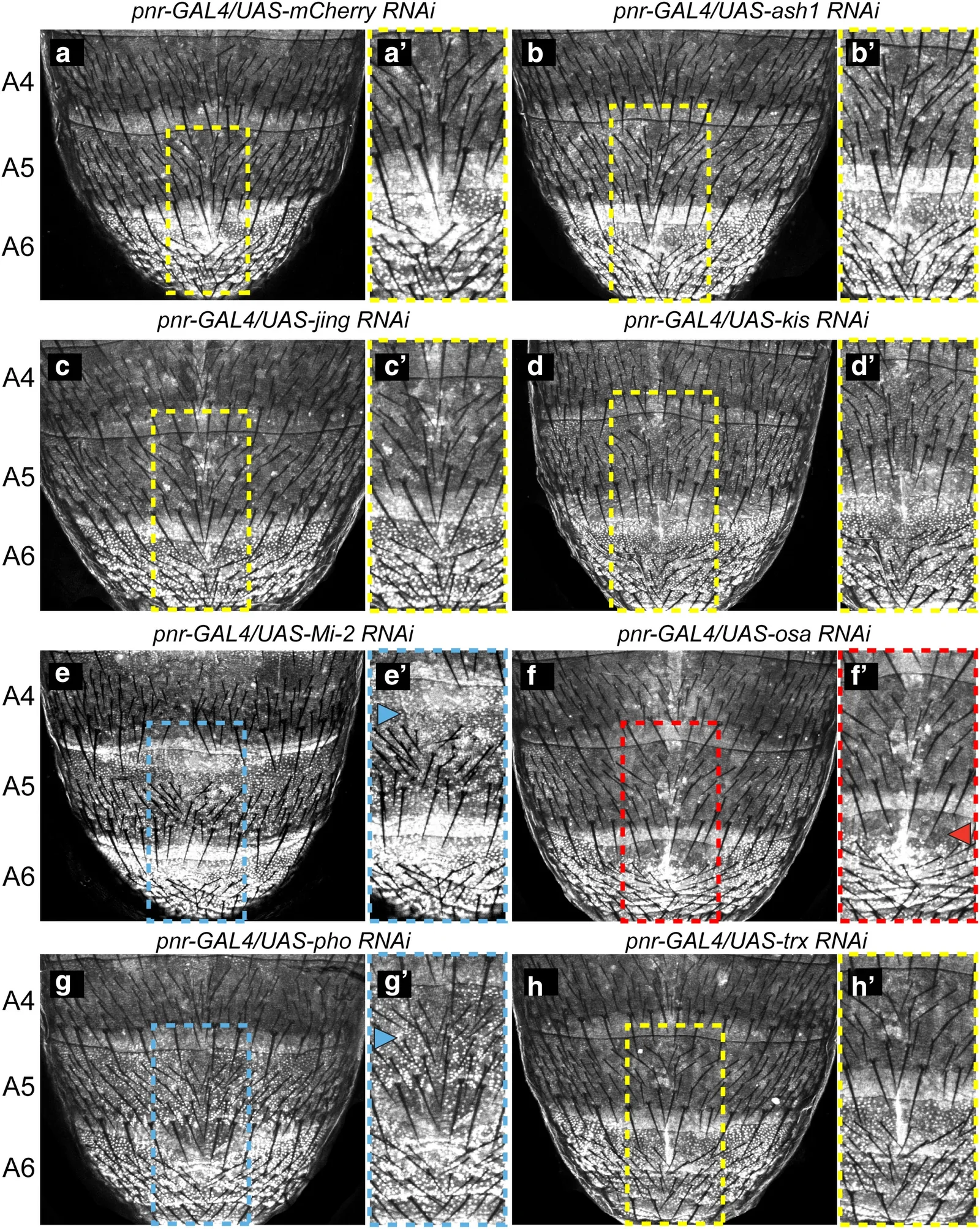
The activities of PcG and TrxG complexes influence the gene expression regulating function of the *bab dimorphic element* CRE in females for key pigmentation GRN genes. a to h) neGFP expressions activated by the *bab dimorphic element* in genetic backgrounds where an RNAi transgene was ectopically expressed by the *pnr-GAL4* driver. a′ to h′) Zoom in images of the dashed rectangle region from the leftward image. The top-half shows expression in the A5 segment, and the bottom-half shows expression in the A6 segment. *D. melanogaster* pupae here are at the 88 hAPF stage. Yellow, blue, and red rectangles respectively indicate regions where neGFP expression was similar, increased, or reduced compared to the *mCherry* RNAi negative control sample.

*Ddc* encodes an enzyme that catalyzes the second step in the pigmentation metabolic pathway (Grover et al. 2018). *Ddc* expression is controlled by the *MEE1* CRE that drives robust expression in the pupal A5 and A6 segments and reduced expression in the more anterior segments of *D. melanogaster*. In reporter transgene assays, this CRE activates greater neGFP expression in male *D. melanogaster* pupal abdomens compared to females (compare Fig. 9 to Supplementary Fig. 28). RNAi for *mCherry* did not perturb this CRE's activity (Fig. 9a and a′). Likewise, RNAi for *jing*, *Mi-2*, *osa*, *pho*, and *trx* had no noticeable effect on this CRE's regulatory activity (Fig. 9). However, RNAi for both *ash1* and *kis* resulted in reduced neGFP expression, notably in the A5 segment (Fig. 9b and d′), driven by the *MEE1* (Fig. 9b and d). This reduction implicates these 2 TrxG proteins as facilitators of this CRE's activity. Interestingly, *ash1* RNAi did not have a similar effect on the CRE's activity in female pupa, and *osa* RNAi caused a noticeable increase in neGFP expression along the dorsal abdomen midline (Supplementary Fig. 28).

**Fig. 9.**
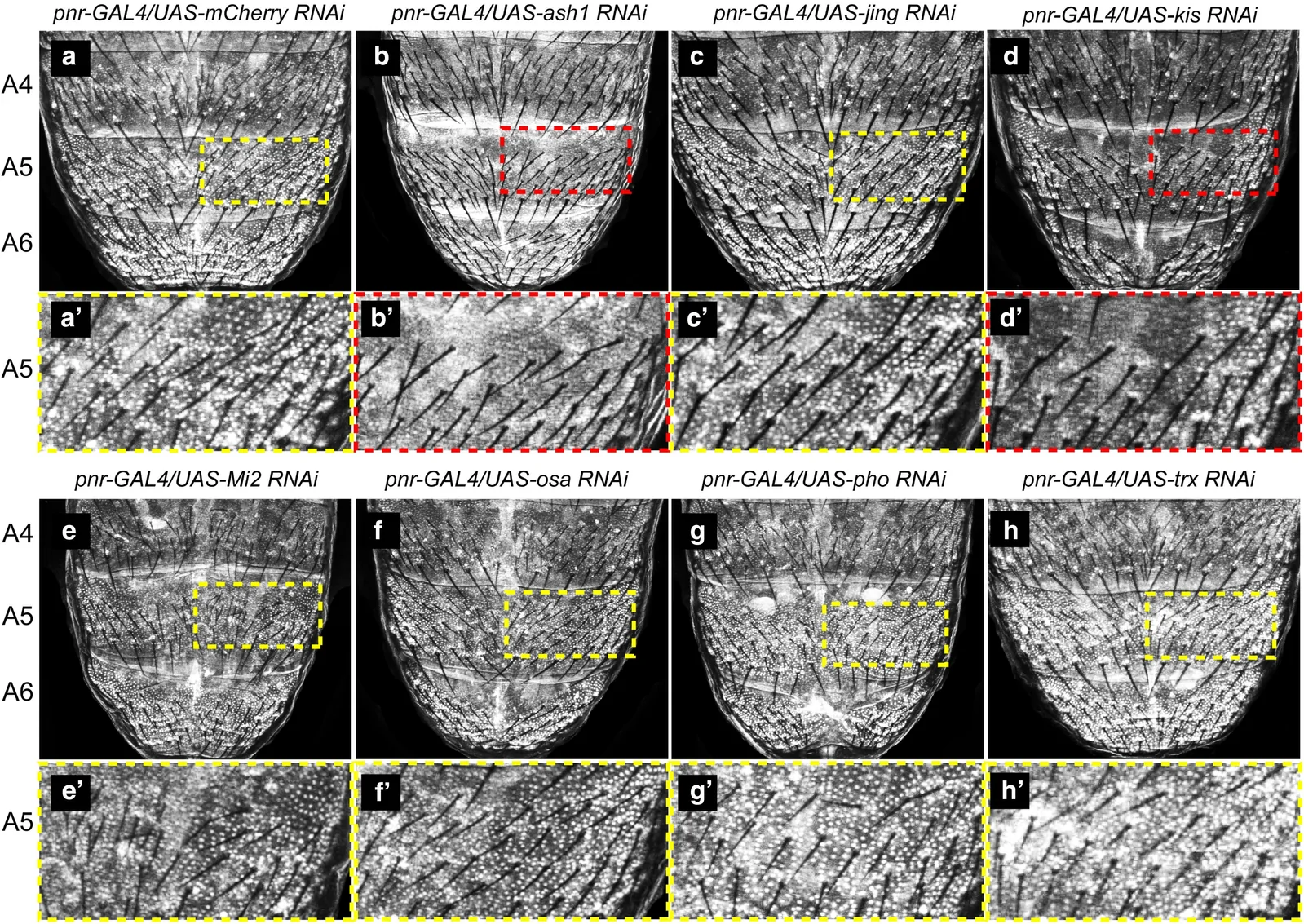
The activities of PcG and TrxG complexes influence the gene expression regulating function of the *Dopa decarboxylase MEE1* CRE in males. a to h) neGFP expressions activated by the *MEE1* in genetic backgrounds where an RNAi transgene was ectopically expressed by the *pnr-GAL4* driver. a′ to h′) Zoom in images of the dashed rectangle region from the A5 segment of the above image. The left side is in the midline where RNAi occurred, and the right side is lateral where RNAi expression was absent. All samples are *D. melanogaster* pupae at the 88 hAPF stage. Yellow and red rectangles indicate regions where neGFP expression was similar or reduced compared to the a) and a′) *mCherry* RNAi negative control sample, respectively.

The Tan enzyme reverses (converts N-β-alanyl dopamine to dopamine) the modification to dopamine caused by the Ebony enzyme (converts dopamine to N-β-alanyl dopamine) to allow for the formation of melanic tergite cuticle (True et al. 2005). The *tan* gene is robustly expressed in the dorsal A5 and A6 segment epidermis of male *D. melanogaster* under the control of the *t_MSE2* CRE (Camino et al. 2015). RNAi for *mCherry* reveals the wild-type pattern of activity for this CRE, with neGFP expression occurring at high levels in the male abdomen (Fig. 10) and very low levels in the female abdomen (Supplementary Fig. 29). RNAi for *ash1*, *jing*, *kis*, *osa*, and *trx* each caused a loss of neGFP expression in the male abdomen of *D. melanogaster* pupa (Fig. 10). On the other hand, RNAi for *Mi-2* resulted in a noticeable increase in neGFP expression in the male A4 segment (Fig. 10e). These results implicate both PcG and TrxG proteins as potentiators of t_MSE2 activity in the male abdomen and the Mi-2 TrxG protein as a repressor. In female pupa, RNAi for *ash1*, *jing*, *osa*, and *trx* resulted in a noticeably lower level of neGFP expression in the A6 segment (Supplementary Fig. 29). More interestingly though was the dramatic increase in neGFP expression when *Mi-2* was targeted by RNAi (Supplementary Fig. 29e and e′). Thus, Mi-2 is a regulator of *tan* expression.

**Fig. 10.**
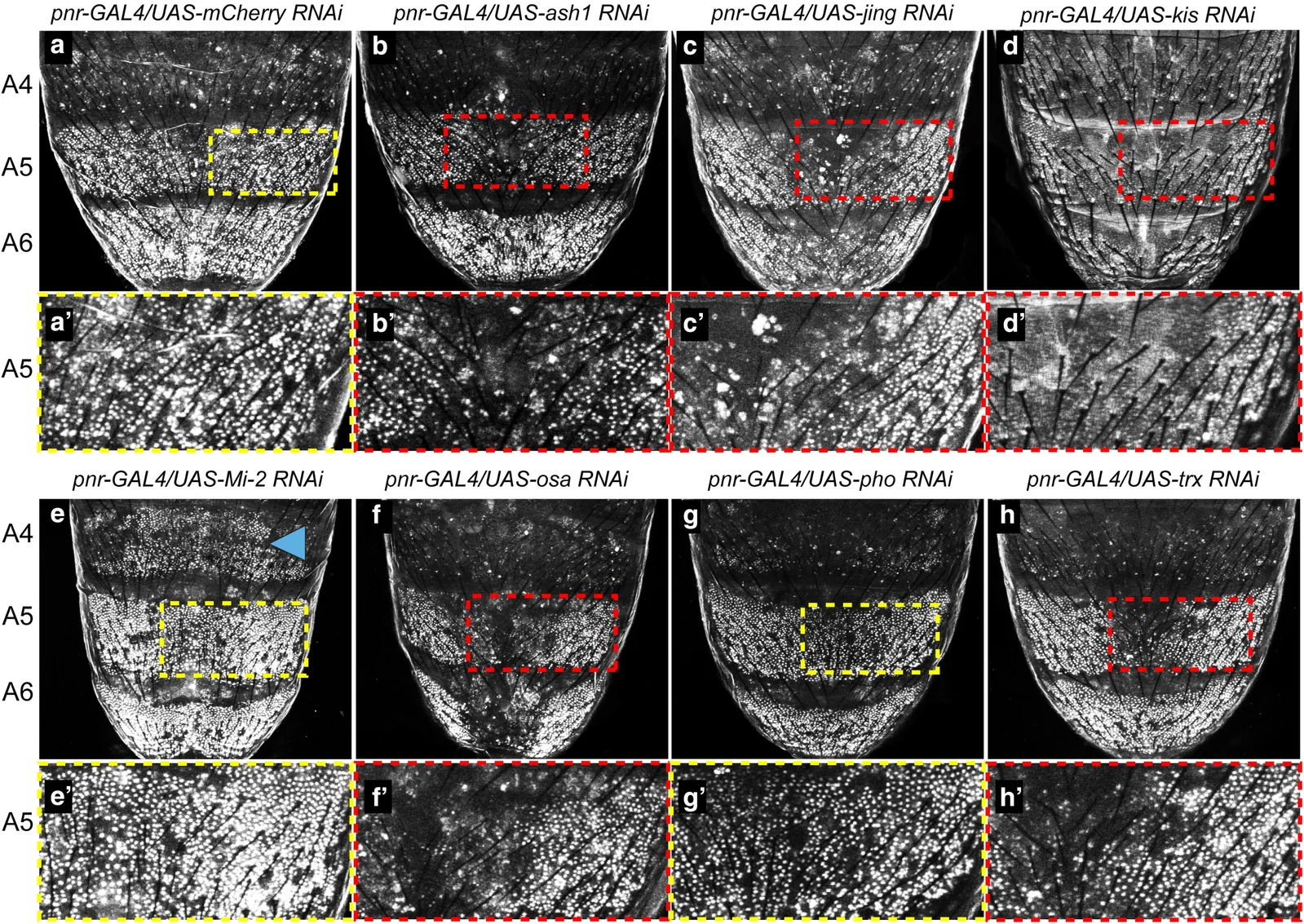
The activities of PcG and TrxG complexes influence the activities of the *tan MSE2 cis*-regulatory element. a to h) neGFP expressions activated by the *t_MSE2* in genetic backgrounds where an *RNAi* transgene was ectopically expressed by the *pnr-GAL4* driver. a′ to h′) Zoom in images of the dashed rectangle region from the A5 segment of the above image. The left side is in the midline where RNAi occurred, and the right side is lateral where RNAi expression was absent. All samples are *D. melanogaster* pupa at the 95 hAPF stage. Yellow and red rectangles respectively indicate regions where neGFP expression was similar or reduced compared to the a) and a′) *mCherry* RNAi negative control sample. e) The blue arrowhead indicates the ectopic neGFP expression that occurs in the A5 male segment following *Mi-2* RNAi.

The *yellow* gene has one of the most dramatic phenotypic effects when mutant, and yet its enzymatic function remains unclear (Hinaux et al. 2018). *yellow* is highly expressed in the male A5 and A6 segment dorsal epidermis starting at or even before 80 hAPF (Camino et al. 2015). This expression pattern is controlled by the *yellow Body Element* CRE (Jeong et al. 2006) with its minimal sequence known as the *BE0.6* CRE (Camino et al. 2015). Compared to an *mCherry* RNAi control (compare Fig. 11a and a′ to Supplementary Fig. 30a and a′), RNAi targeting *ash1*, *jing*, *kis*, *Mi-2*, *pho*, *Pc*, and *trx* resulted in no noteworthy difference in neGFP expression in males and females (Fig. 11; Supplementary Fig. 30). However, reduced expression was observed in the male abdomen when *osa* was targeted by RNAi (Fig. 11f and f′), implicating this TrxG component as an upstream factor that facilitates this CRE's activity. It is important for future studies to resolve whether the influences of these PcG and TrxG proteins on the GRN CREs are mediated by direct or indirect mechanisms.

**Fig. 11.**
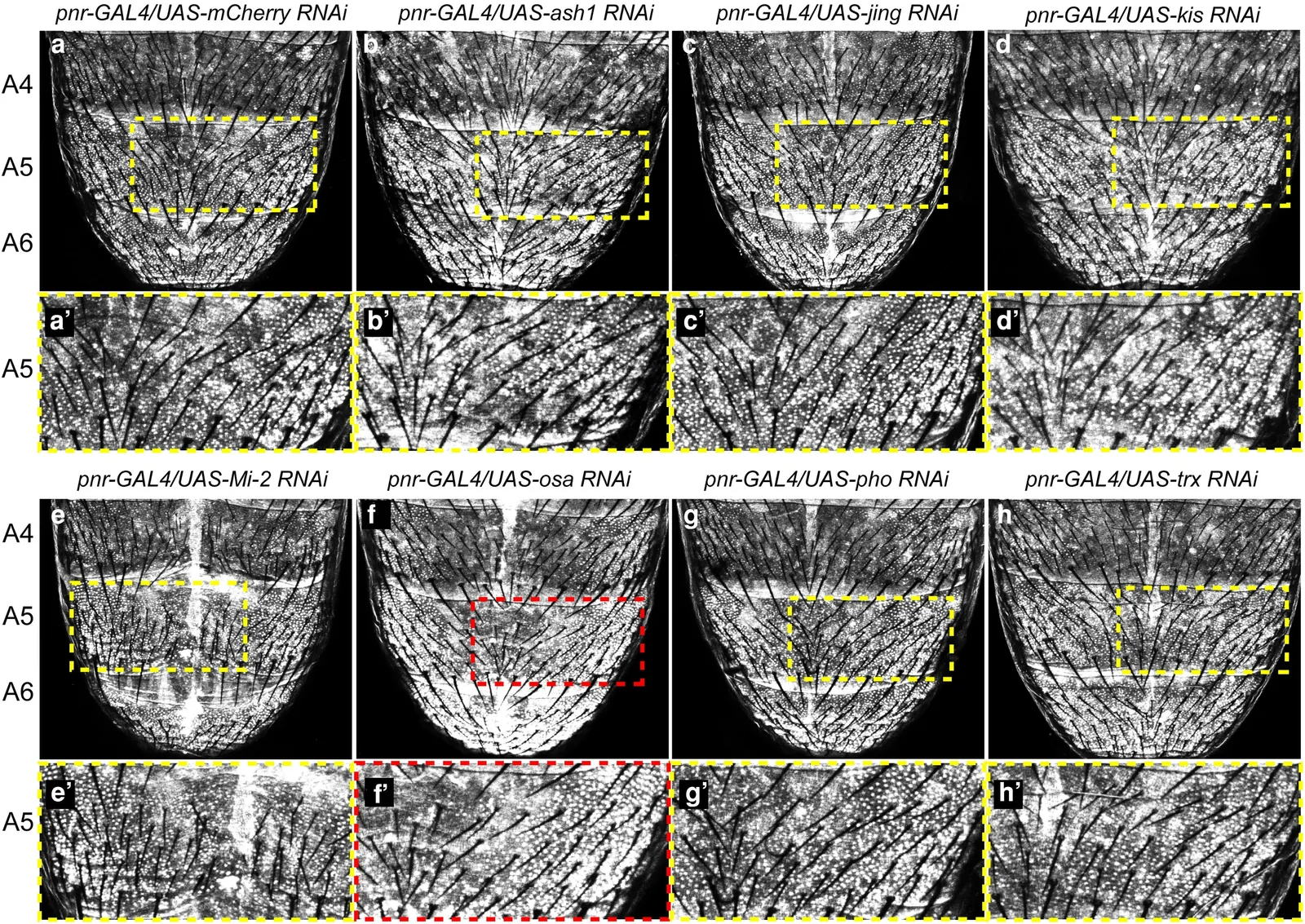
The activities of PcG and TrxG complexes influence the activities of the *yellow BE0.6 cis*-regulatory element in males. a to h) neGFP expressions activated by the *yBE0.6* in genetic backgrounds where an *RNAi* transgene was ectopically expressed by the *pnr-GAL4* driver. a′ to h′) Zoom in images of the dashed rectangle region from the A5 segment of the above image. The left side is in the midline where RNAi occurred, and the right side is lateral where RNAi expression was absent. All samples are *D. melanogaster* pupa at the 88 hAPF stage. Yellow and red rectangles respectively indicate regions where neGFP expression was similar or reduced compared to the a) and a′) *mCherry* RNAi negative control sample.

## Discussion

Here, we performed a robust characterization of the differing roles played by PcG and TrxG genes in the development of the sexually dimorphic pattern of abdomen tergite pigmentation for the fruit fly species *D. melanogaster*. The results show how subsets of these chromatin-modifying genes influence the GRN's activity through unique means to arrive at both spatial and sex-specific pigmentation patterns (Fig. 12). Indeed, no 2 treatments affected pigmentation in exactly the same way. Notably, the absence of Abd-B expression in the anterior abdomen remains under the influence of the PRC1 and PRC2 PcG complexes (Fig. 12; Supplementary Figs. 31 and 32), as shown previously for embryonic and larval stages of development (Busturia and Bienz 1993; Busturia et al. 1997). In the posterior abdomen where Abd-B is expressed, the repressive roles of PRC1 and PRC2 are absent. This absence is perhaps due to the influence of the H3K36 demethylase activity of Ash1 of the TrxG. The robust expression of the pigmentation realizator genes through their CREs was shown to rely upon unique contributions of differing TrxG proteins (Supplementary Figs. 31 and 32). The absence of broad melanic pigmentation from the posterior female abdomen is prominently shaped by the Bab transcription factors by their repressive action on the expression of several realizator genes. Our results show that Bab expression in females requires the pupal-stage activity of the nucleosome remodeling factor Mi-2 (Fig. 12; Supplementary Fig. 31). A reduction of Mi-2 expression led to both ectopic Abd-B expression and tergite pigmentation, revealing a role for Mi-2 in shaping this GRN's activity and pigmentation phenotype in the anterior abdomen as well.

**Fig. 12.**
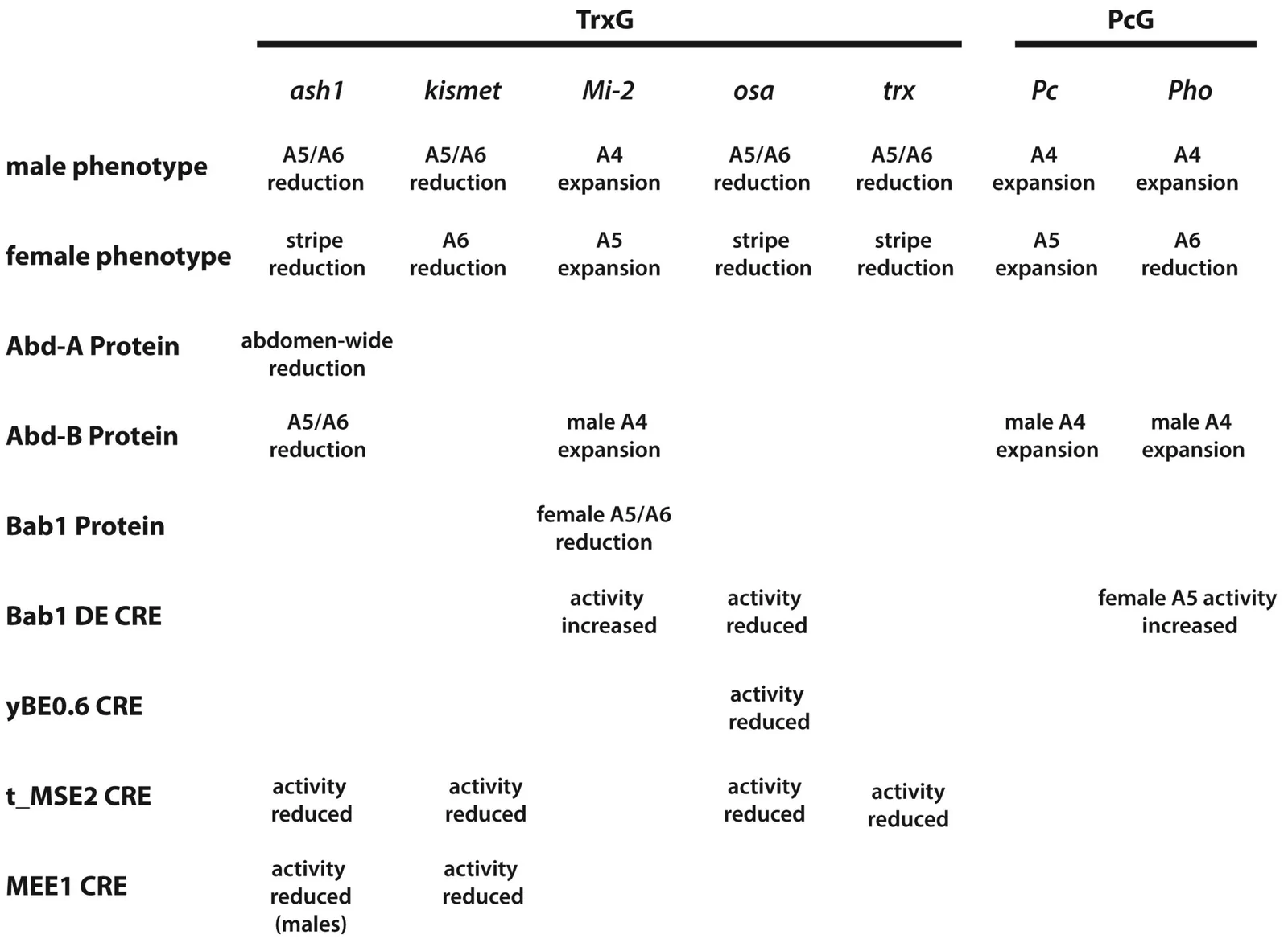
Summary of key results, indicating the phenotypes in males and females as well as TF protein expression and CRE reporter phenotypes of RNAi driven by *pnr-GAL4* for 2 PcG genes and 5 TrxG genes. Paradoxically, *Mi-2* RNAi resulted in reduced Bab1 expression in the posterior female abdomen, while it elicited ectopic reporter gene expression driven by the *dimorphic element* CRE that controls female-specific *bab* expression. This indicates that the overall effect of *Mi-2* on *bab* expression involves additional elements beyond this one CRE. The outcomes listed for *Pc* were those elicited by the use of the *UAS-Pc* RNAi line #33964.

### The roles of chromatin modification and remodeling across a GRN

One of the earliest sources of patterning in the abdominal pigmentation network is Abd-B, which is activated in embryonic tissues and maintained into postembryonic development (Sánchez-Herrero and Crosby 1988; Kopp and Duncan 2002). It has been known for decades that the expression of *Abd-B* is under the genetic and molecular control of PcG and TrxG genes and their encoded proteins acting on CREs within this gene locus (Celniker et al. 1990). The *Mcp* region of the *Abd-B* locus has been shown to function as a PRE that silences Abd-B expression during embryonic and larval development in the more anterior A4 segment (Sánchez-Herrero 1991; Busturia and Bienz 1993; Busturia et al. 1997). This PRE possesses several binding sites for Pho (Busturia et al. 2001), which likely recruits the PRC1 and PRC2 complexes to this element and thereby silences *Abd-B* expression. PcG silencing of Abd-B expression is not limited to embryonic and larval stages, as it has been shown to occur in the adult testis as well (Zhang et al. 2017).

The maintenance of *Abd-B* and other *Drosophila Hox* gene expression was shown to require the input of the *trithorax* gene, the founding member of the TrxG group (Breen and Harte 1993). The expression-promoting effect of *trx* is required during larval stages too (Ingham 1981). Thus, it seemed logical that Trx would also sustain Hox expression during the metamorphic pupal stage of development. In line with this reasoning, the wild-type pattern of *D. melanogaster* tergite pigmentation requires *trx* (Weinstein et al. 2023). Surprisingly, a reduction in *trx* expression by RNAi during pupal development did not noticeably alter Abd-B nor Bab1 expression. This loss in Trx activity, however, did result in the abnormal activity of the CREs controlling the expression of the *tan* pigmentation realizator gene. This seems to indicate that other factors are responsible for the maintenance of Abd-B into pupal development, and at these stages, Trx function appears to directly regulate realizator genes of the GRN.

In this study, we mapped the sites of TrxG gene function within the tergite pigmentation GRN. Most of the TrxG genes influenced the activities of realizator gene CREs. The TrxG gene *ash1*, however, was additionally shown to influence the expression of Abd-B and Abd-A. While the TrxG genes *osa*, *kis*, and *trx* had striking tergite pigmentation phenotypes in response to pupal-stage RNAi, Abd-A, Abd-B, and Bab1 expression was unperturbed. Moreover, RNAi for *Mi-2* resulted in females with a conspicuous male-like pattern of tergite pigmentation. While some degree of ectopic Abd-B expression occurred, reduced Mi-2 expression led to a loss of Bab1 expression and the ectopic activity of the *tan* gene CRE (a target of Bab repression) in females. One inconsistency among our results is the expanded activity of the *bab dimorphic element* (*de*) CRE activity despite a marked reduction of Bab1 expression upon *Mi-2* RNAi. This result may be due to cooperative CRE interactions that are not captured in standard reporter transgene assays, or this result may reflect a broader failure of enhancer–promoter communication in the Mi-2 RNAi treatment. Collectively, these results draw attention to how the PcG proteins and complexes continuously maintain the spatial boundary of Abd-B expression during pupal development, while the TrxG proteins have activities that map to multiple GRN tiers, including the CREs of the most terminal realizator genes.

### Finding the genomic targets of PcG and TrxG function

Our RNAi experiments implicate pupal development as another critical stage of PcG and TrxG function and present provocative targets of PcG and TrxG molecular function. However, much difficult work remains to be done. In the case of the TrxG proteins, it is possible that their effects reflect the action of an intermediary upstream regulator whose encoded protein is the actual direct regulator of realizator gene expression. Alternatively, the TrxG proteins and their molecular complexes may act through their recruitment to these realizator gene CREs. In either case, it is important to identify what factor recruits the particular TrxG proteins to particular CREs.

In the formulation of our GRN models (Fig. 12 to 14), we worked under the assumption that the PcG and TrxG proteins are more or less ubiquitously expressed in anterior and posterior abdominal segments, similarly between males and females, and present during early, middle, and late pupal stages. This could be spectacularly wrong if these chromatin modifiers have more complex patterns of expression. For instance, RNAi for *osa* and *Mi-2* had astounding effects on pigmentation when paired with a GAL4 driver that was expressed early during pupal development. This outcome could be due to an activity of these factors that occur early in development. It appears Pho brings PcG complexes to the Abd-B *Mcp* silencer. If Pho was not expressed in the posterior abdomen, it would explain why Abd-B is able to avoid being silenced in these segments. Temporal differences in the activity of chromatin modification complexes should not be overlooked, as a precedent exists in the literature. Specifically, the DotCom H3K79 methylase complex was shown to play opposite roles in *D. melanogaster* abdomen pigmentation during early and late developmental stages (Narbey et al. 2024).

**Fig. 13.**
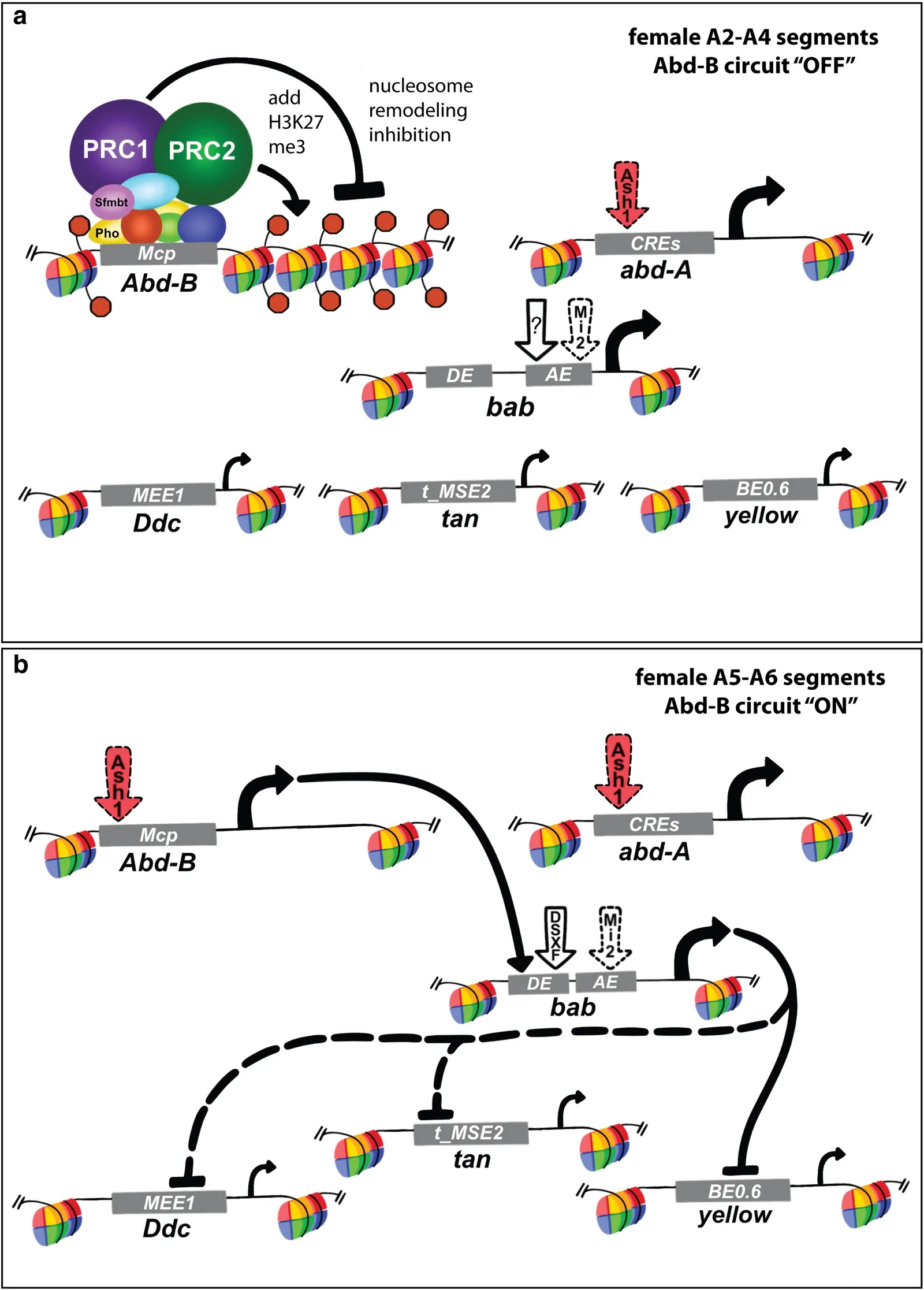
A chromatin landscape model for the pigmentation GRN in the *D. melanogaster* female abdomen. Melanic pigmentation requires the robust activation of the *Ddc*, *tan*, and *yellow* realizator genes through the activity of these genes’ CREs. a) In the anterior abdomen segments (A2 to A4), abdomen realizator gene expressions are minimal due to the absence of the positive input of Abd-B. Abd-B expression in these segments is turned “OFF” through the activity of the PRC1 and PRC2 complexes creating a repressive chromatin landscape by histone H3 tri-methylation (red stop signs) and an inhibition on chromatin remodeling. b) In the posterior abdomen segments (A5-A6), Abd-B chromatin landscape is favorable for its expression. This allows Abd-B to activate the expression of *bab* in combination with the female isoform of the Dsx (DsxF) transcription factor. Bab transcription factors repress the pigmentation realizator genes. Solid connecting arrows indicate direct regulation where Abd-B or Bab bind to sites within a CRE, while dashed connecting arrows indicate situations where regulation has not been shown to be direct. The colored arrows with a dashed outline indicate CREs that have been shown to be sensitive to a TrxG protein, though it remains uncertain whether this effect is direct or indirect.

**Fig. 14.**
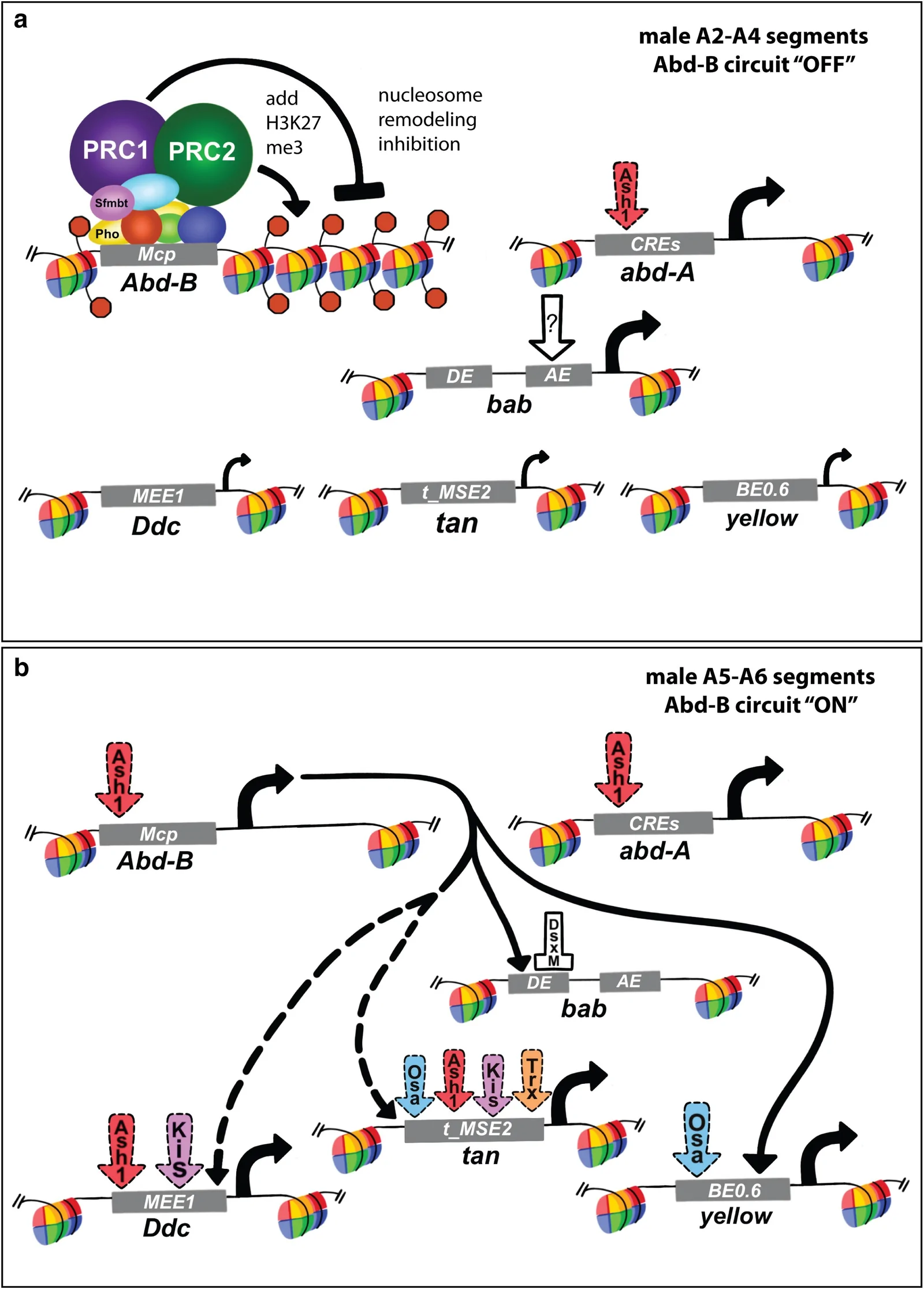
A chromatin landscape model for the pigmentation GRN in the *D. melanogaster* male abdomen. Melanic pigmentation requires the robust activation of the *Ddc*, *tan*, and *yellow* realizator genes through the activity of these genes’ CREs. a) In the anterior abdomen segments (A2 to A4), abdomen realizator gene expressions are minimal due to the absence of the positive input of Abd-B. Abd-B expression in these segments is turned “OFF” through the activity of the PRC1 and PRC2 complexes creating a repressive chromatin landscape by histone H3 tri-methylation (red stop signs) and an inhibition on chromatin remodeling. b) In the posterior abdomen segments (A5-A6), the Abd-B chromatin landscape is favorable for its expression. This allows Abd-B to activate the expression of its target genes, including the realizators. Solid connecting arrows indicate direct regulation where Abd-B binds to sites with the CRE, while dashed connecting arrows indicate situations where Abd-B regulation has not been shown to be direct. The colored arrows with a dashed outline indicate CREs that have been shown to be sensitive to a TrxG protein, though it remains uncertain whether this effect is direct or indirect.

### Incorporating PcG and TrxG into the evo-devo paradigm

The tergite pigmentation pattern of *D. melanogaster* is a compelling model to understand how GRNs can produce spatial and sex-specific patterns of gene expression and their phenotypic manifestation. However, the full potential of this model trait is realized in the context of comparative studies of trait evolution. The prevailing thought is that dimorphic tergite pigmentation originated from a monomorphic ancestral state, and the number of melanic male tergites has expanded and contracted in various lineages (Rebeiz and Williams 2017). This dimorphic pattern of pigmentation has been lost on several occasions too (Jeong et al. 2006; Camino et al. 2015). It thus stands to reason that the PcG and TrxG factors studied here either played direct or indirect roles in these transitions. Were these factors always expressed in the abdomen, or did derived functions of these genes evolve?

Our finding that the *tan MSE2* CRE is dependent upon multiple TrxG complex members, while other co-expressed genes such as *yellow* are not, raises the exciting possibility that a Trithorax response element evolved specifically in the t_MSE2 region. Of note, our past work identified 2 regions by scanning mutagenesis that were required for *MSE2* activity (Camino et al. 2015). Binding site mutations within this region failed to identify activating transcription factor binding sites, though did identify a role for Abd-A and Homothorax (Hth) in repressing the anterior boundary of *MSE2* activity. Further work on this CRE may identify how a Trithorax-response element evolved in the context of a spatially patterned GRN. Finding such novel elements might reveal general principles as to how chromatin modifiers contribute to trait formation and evolution.

## Data Availability

*Drosophila melanogaster* reporter transgene strains and plasmids are available upon request. All data analyses and images are contained in this manuscript and its supplemental material. Supplemental materials are available via FigShare (https://doi.org/10.6084/m9.figshare.33000008). These materials include 32 supplementary figures, a supplementary figure legends document, Supplementary Document 1 which provides the sequence and annotations for a novel *GAL4* transgene vector, and Supplementary Document 2 that provides a description of the supplementary results.
